# Increased CD44 Expression in Endothelial Cells Induced by Advanced Glycation End Products Leads to Insufficient Maturation of Angiogenesis

**DOI:** 10.1111/jcmm.71088

**Published:** 2026-03-16

**Authors:** Xiaoxia Huang, Zhuanhua Liu, Jiaqing Hu, Bingyu Li, Zhenfeng Chen, Tairan Zeng, Xing Zhou, Ruimin Lu, Wenyan Deng, Wendong Zhou, Qiaobing Huang

**Affiliations:** ^1^ Department of Pathophysiology Southern Medical University Guangzhou China; ^2^ Department of Cardiology The Eighth Affiliated Hospital of Southern Medical University Foshan China

**Keywords:** advanced glycation end products, angiogenesis, basement membrane, CD44, matrix metalloproteinases 9

## Abstract

Pathological angiogenesis occurs in various diseases, including tumours, diabetes and wound healing. The endothelial cells lining these aberrant neovessels exhibit abnormal morphology, with loosely attached or absent pericytes and the basement membrane (BM) is often disrupted. Advanced glycation end products (AGEs) promote pathological angiogenesis in diabetic vascular complications, but whether the vascular BM structure is altered and the underlying molecular mechanisms remain unclear. By analysing single‐cell RNA sequencing data (GSE204880) from the oxygen‐induced retinopathy mouse model, this study found heterogeneous expression of type IV collagen (Col‐IV) and laminin in endothelial cells, with enrichment of the AGE‐RAGE pathway and BM‐related pathways. Experimental results demonstrated that AGEs induce abnormal distribution of Col‐IV in vascular BM, along with increased Col‐IV levels in mouse serum and endothelial‐pericyte co‐culture supernatants, suggesting excessive BM degradation. Additionally, AGEs upregulated CD44 and matrix metalloproteinases 9 (MMP9) protein levels in retinal tissues and endothelial cells. Knockout/knockdown of CD44 or inhibition of MMP9, can both significantly alleviate BM structural disruption and abnormal angiogenesis. The down‐regulation of CD44 expression or application of γ‐secretase inhibitor DAPT attenuated AGEs‐promoted MMP9 expression and secretion. Moreover, AGEs facilitated β‐catenin nuclear translocation and its interaction with TCF4. When this interaction was blocked by LF3, AGEs‐induced CD44 upregulation was reduced, and pathological angiogenesis and BM abnormalities were partially restored. These findings suggest that AGEs upregulate endothelial CD44 expression via β‐catenin/TCF4 signalling pathway, which in turn promotes MMP9‐mediated excessive degradation of BM components, leading to structural disorganisation and impaired vascular maturation in pathological angiogenesis.

## 1 Introduction

Diabetes mellitus represents a significant global health challenge, emerging as one of the most rapidly escalating health threats of the 21st century. According to the report of the International Diabetes Federation in 2025, the global prevalence of diabetes among adults aged 20–79 years reached 589 million in 2024, and it is estimated to increase to 853 million by 2050 [1]. Diabetes‐related fatalities exceeded 3.4 million, accounting for 9.3% of global deaths in 2024 [1]. The high mortality associated with diabetes is not due to hyperglycemia but to the diverse vascular complications it causes [2]. Patients with diabetes are susceptible to life‐threatening complications, including macrovascular diseases, such as coronary artery disease, stroke and peripheral artery disease, as well as microvascular complications including retinopathy, neuropathy and nephropathy [3]. Diabetic retinopathy (DR), the most prevalent microvascular complication of diabetes [4], is a leading cause of blindness in developed countries [5].

Advanced glycation end products (AGEs) are generated within a persistent hyperglycemic environment, arising from the formation and accumulation of cytotoxic byproducts, such as carbonyl compounds, and subsequent non‐enzymatic reactions with proteins, lipids and nucleic acids [6, 7]. Studies have shown that serum AGE levels are significantly increased in diabetic patients [8] and are associated with the severity of diabetic complications [9, 10]. Despite glycemic control, many diabetic patients continue to experience severe vascular complications, a phenomenon linked to cellular metabolic memory induced by sustained hyperglycemia [11]. Persistent hyperglycemia epigenetically modifies cellular gene expression profiles, establishing metabolic memory that sustains diabetic complication progression even post‐glycemic normalisation, with potential transgenerational transmission [12]. It has been pointed out that the legacy effect of hyperglycemia is related to the accumulation of AGEs [11, 12]. Therefore, elucidating the pathogenic mechanisms of AGEs in diabetes and its complications is essential for their therapeutic interventions.

Angiogenesis, the formation of new blood vessels from pre‐existing vascular system, involves the proliferation and migration of endothelial cells and subsequent pericyte recruitment and basement membrane (BM) establishment, which is crucial for the stabilisation of vascular structure and its normal physiological function. However, angiogenesis also underlies pathological processes in diseases such as tumours and atherosclerosis, and is also a key feature of diabetic microvascular complications, especially in proliferative DR [13]. Our previous studies have demonstrated that AGEs promote angiogenesis both in vitro and in vivo [14, 15]. Furthermore, we observed impaired endothelial cell adhesion [16] and a reduction in pericyte coverage [14]. Since BM formation relies on endothelial cell‐pericyte interactions, which are crucial for vascular maturation, we further demonstrated that there were abnormalities in BM of these aberrant neovessels [17]. However, the underlying molecular mechanisms remain to be elucidated.

CD44, a ubiquitously expressed cell surface glycoprotein, is a well‐established marker for cancer stem cells. Recent investigations have implicated the effect of CD44 in the regulation of tissue glucose metabolism and the exacerbation of insulin resistance, thereby playing a significant role in metabolic disorders such as obesity and diabetes [18]. Our prior studies have demonstrated that the AGEs in diabetes promote CD44 expression in pericytes, thereby enhancing pericyte migration and detachment [19], which subsequently leads to impaired neovascular maturation and the development of retinopathy. During angiogenesis, endothelial cells recruit pericytes to establish heterotypic adhesive junctions, and together, they secrete proteins to form the BM structure. CD44 is involved in both cell‐ECM interactions and cell–cell communication. Whether CD44 in endothelial cells participates in AGEs‐induced neovascular immaturity remains to be elucidated.

Here, we investigated the relationship between endothelial CD44 and abnormal BM structure in AGEs‐induced angiogenesis, providing a rationale and novel therapeutic targets for the treatment of diabetic vascular complications such as DR.

## 2 Materials and Methods

### 2.1 Acquisition and Analysis of Transcriptome Sequencing Data

Single‐cell RNA sequencing (scRNA‐seq) data from retinal tissue of oxygen‐induced retinopathy (OIR) mice were obtained from the GEO database (GSE204880) [20]. Quality control of the cells was performed based on the proportion of mitochondrial genes per cell (mitochondrial gene < 30%) and the number of detected genes (cells with 200–5000 genes). Gene expression data were normalised, and cell cluster structure was visualised using UMAP non‐linear dimensionality reduction, followed by cell clustering using the Leiden algorithm. Cell clusters were annotated using known marker gene expression profiles, and the expression of type IV collagen (Col ‐ IV) and laminin (LN), the main components of the BM, was analysed in endothelial cell clusters. KEGG pathway analysis and GO enrichment of characteristic genes were performed on endothelial cell clusters using cluster Profiler R.

Transcriptome data from OIR mouse retinal tissue were derived from the study by Binet et al. [21]. Gene Set Enrichment Analysis (GSEA) pathway analysis was performed on all genes, and genes in the BM assembly pathway were selected and visualised by heatmaps (heatmap, version 4.4.3) and volcano map (ggplot2, version 3.5.1) produced by R (version 4.5.1). The genes with padj. < 0.01 and log2FC > 0 were considered as differentially expressed genes.

### 2.2 AGEs Preparation

As previously reported [22], AGEs were prepared in vitro via the glycation method under sterile conditions. A PBS solution (pH 7.4) containing 50 g/L bovine serum albumin (BSA) and 500 mM D‐glucose was prepared. A PBS solution containing only BSA served as a control. Both solutions were incubated at 37°C in the dark. After 8 weeks, the incubation solutions were concentrated and purified. AGE‐specific fluorescence was measured using ratio fluorescence spectrophotometry (excitation, 370 nm; emission, 440 nm). Endotoxin contents in both AGEs and control solutions were assessed using the Limulus amebocyte lysate assay, and a concentration below 500 U/L was considered acceptable.

### 2.3 Experimental Animals

Wild‐type (WT) C57BL/6J mice, aged 7–8 weeks, and pregnant mice were obtained from the Experimental Animal Center of Southern Medical University. CD44 knockout mice were purchased from Cyagen Biosciences (China, S‐KO‐01419) and were bred and genotyped according to the company's instructions (Primers for genotyping are shown in Table S1). All animals were housed in a specific pathogen‐free environment at 23°C–25°C with 50% ± 5% humidity, a 12‐h light/dark cycle, and provided with ad libitum access to chow and water. All animal experiments were approved by the Animal Care and Ethics Committee of Southern Medical University and adhered to the ethical guidelines for animal research.

Based on our previously published article [14], this study employed an AGE‐treated murine angiogenesis model. Neonatal pups received daily intragastric administration of AGEs at 10 mg/kg for 7 days. Adult male mice, aged 7–8 weeks, were administered AGEs via intraperitoneal injection at 10 mg/kg daily for either 1 or 6 months to simulate the chronic accumulation of AGEs observed in diabetes. At the end of the experiment, mice were anaesthetised (isoflurane for neonatal mice, pentobarbital sodium for adult mice), followed by blood collection from the orbital venous plexus and eyeball removal for further experiments. Given the lower incidence of diabetes in females, which may be attributed to the protective effect of oestrogen [23, 24], only male mice were utilised in these experiments.

### 2.4 Cell Culture and AGEs Treatment

Human umbilical vein endothelial cells (HUVECs) were obtained from ScienCell (USA, Cat#8000) and cultured in endothelial cell medium supplemented with 5% fetal bovine serum (FBS), 1% endothelial cell growth supplement and 1% penicillin/streptomycin solution. Human brain vascular pericytes (HBVPs) were purchased from Wuhan Sunncell Biotechnology Co. Ltd. (China, Cat#SNP‐H182) and maintained in pericyte‐specific medium containing 2% FBS and 1% pericyte growth factor.

Based on our preliminary dose–response studies (50–200 μg/mL) [25] and supporting literature [26], 100 μg/mL AGEs were used to stimulate HUVECs under serum‐restricted culture conditions.

### 2.5 Matrigel Co‐Culture Tube Formation Assay

The Matrigel was thawed overnight at 4°C. Subsequently, 50 μL of Matrigel was evenly distributed onto 10 mm glass‐bottom dishes (Cellvis, Cat# D35‐10‐1‐N) and polymerised at 37°C for 30 min. A mixture of 3 × 10^4^ HUVECs and 6 × 10^3^ HBVPs (at the ratio of 5:1), suspended in 150 μL of culture medium, was seeded onto the dishes. Images of tube formation were captured 8–10 h post‐seeding, and the total tube length and number of branch points were quantified using AngioTool software. The co‐culture supernatants were collected, and the tubes were subsequently processed for immunofluorescence staining.

### 2.6 Immunofluorescence Staining

For retinal immunofluorescence in mice, staining was performed as described in previous studies [27]. Briefly, mouse eyeballs were harvested following euthanasia and fixed in 4% paraformaldehyde at 4°C for 2 h. Following fixation, the eyeballs were washed five times in ice‐cold 1× PBS, and the retinas were dissected, with four radial incisions made around the periphery. Retinas were blocked overnight at 4°C in a blocking solution of 1% BSA and 0.3% TritonX‐100 in PBS. Primary antibodies against Col‐IV or LN were diluted in blocking solution containing 2% goat serum and incubated with the retinas overnight at 4°C. The retinas were washed five times with a washing buffer consisting of a 1:1 mixture of blocking solution and PBS. Secondary antibodies, goat‐derived and conjugated to Alexa Fluor 594, were diluted in antibody dilution buffer and incubated at room temperature for 2 h, protected from light. After washing with washing buffer, nuclei were counter‐stained with DAPI. The retinas were mounted on slides (with the inner retinal vasculature facing upwards) and cover‐slipped with an antifade mounting medium.

For HUVECs alone or HUVECs co‐cultured with HBVPs in vitro experiments, staining was performed following the subsequent procedure. At the end of the experiment, cells were washed three times with 1× PBS and subsequently fixed with 4% paraformaldehyde for 10–15 min. Then cells were permeabilised with 1× PBS containing 0.25% Triton X‐100. After blocking with goat serum for 1 h, primary antibodies were incubated overnight at 4°C (antibody details and usage concentrations are listed in Table S2). The following day, cells were washed three times with 1× PBS and incubated with Alexa Fluor 488 or 594‐conjugated secondary antibodies for 2 h at room temperature. Following 1× PBS washes, nuclei were counter‐stained with DAPI, and images were captured via laser scanning confocal microscopy (Zeiss LSM880, Germany).

### 2.7 Adenovirus Transfection

The CD44‐interfering adenovirus (shCD44, Cat#111753–1) and its negative control virus (NC, Cat#CON098) were both obtained from GeneChem Co. Ltd. (Shanghai, China). HUVECs were transduced with the adenoviruses at approximately 40% confluency using serum‐reduced medium. After 8 h, the medium was replaced with complete medium, and the cells were cultured for an additional 48 h. Subsequently, cellular RNA and total protein were extracted to validate the interference efficiency via qPCR and Western blot analysis.

### 2.8 Co‐Immunoprecipitation (Co‐IP)

HUVECs were lysed using IP lysis buffer (Beyotime, China, Cat#P0013) containing PMSF, a protease inhibitor cocktail and a phosphatase inhibitor cocktail. A portion of the lysate was retained as the Input group, while the remainder was incubated with an anti‐β‐catenin IP antibody overnight at 4°C. The following day, Protein A + G Agarose (Beyotime, China) was added, and the mixture was incubated for 3 h at 4°C. After washing with PBS, the immunoprecipitates were resuspended in 1× protein loading buffer and subjected to Western blot analysis.

### 2.9 Western Blot

Total protein from tissues and cells was lysed in RIPA buffer containing PMSF, protease inhibitor cocktail and phosphatase inhibitor cocktail. Following complete lysis, samples were centrifuged at 12,000× *g* for 15 min at 4°C, and the supernatant was collected as total protein. Cytoplasmic and nuclear proteins were extracted sequentially using a commercial kit (Bestbio, China, Cat#BB‐3102) according to the manufacturer's instructions. Briefly, cells were lysed in Protein Extraction Buffer A, followed by centrifugation at 2000× *g* for 5 min at 4°C to collect cytoplasmic proteins. The nuclear pellet was then resuspended in Protein Extraction Buffer B, sonicated and incubated on ice. Finally, nuclear proteins were isolated by centrifugation at 12,000× *g* for 10 min at 4°C. Protein concentrations of all samples were determined by the BCA assay. Protein samples (30–50 μg per well) were separated by 10% SDS‐PAGE and transferred to polyvinyl difluoride membranes. Membranes were blocked with 5% skimmed milk or 5% BSA at room temperature for 1 h, followed by overnight incubation at 4°C with specific primary antibodies (antibody details and usage concentrations are listed in Table S2). The following day, membranes were washed with 0.1% TBST and incubated with HRP‐conjugated secondary antibodies at room temperature for 1–2 h. Proteins were visualised using ECL chemiluminescence and analysed for quantification using ImageJ software.

### 2.10 Enzyme‐Linked Immunosorbent Assay (ELISA)

Col‐IV, LN and matrix metalloproteinases 9 (MMP9) levels in mouse serum and endothelial cell‐pericyte co‐culture supernatants were quantified using ELISA kits, according to the manufacturer's protocol. Briefly, serum or co‐culture supernatant was added to antibody‐coated microplates and incubated. Following incubation, biotinylated antibodies and HRP‐conjugated enzyme substrate were sequentially added. After incubation with the chromogenic substrate, absorbance was read at 450 nm using a microplate reader. Sample concentrations were determined by a standard curve generated from known concentrations of standards.

### 2.11 Statistical Analysis

All data were analysed using GraphPad Prism 8.0 software and presented as mean ± SEM. For comparison of data between two groups, Student's *t*‐test was conducted. For comparison of data among more than two groups, one‐way analysis of variance (ANOVA) was used with post hoc analysis using Tukey or Tamhane's test when there was only one independent variable, and two‐way ANOVA with Sidak post hoc test was used for analyses involving two independent variables. *P* value < 0.05 was considered statistically significant.

## 3 Results

### 3.1 AGEs Promote Angiogenesis Both In Vitro and In Vivo, Which Is Associated With Abnormal BM Structure

**FIGURE 1:**
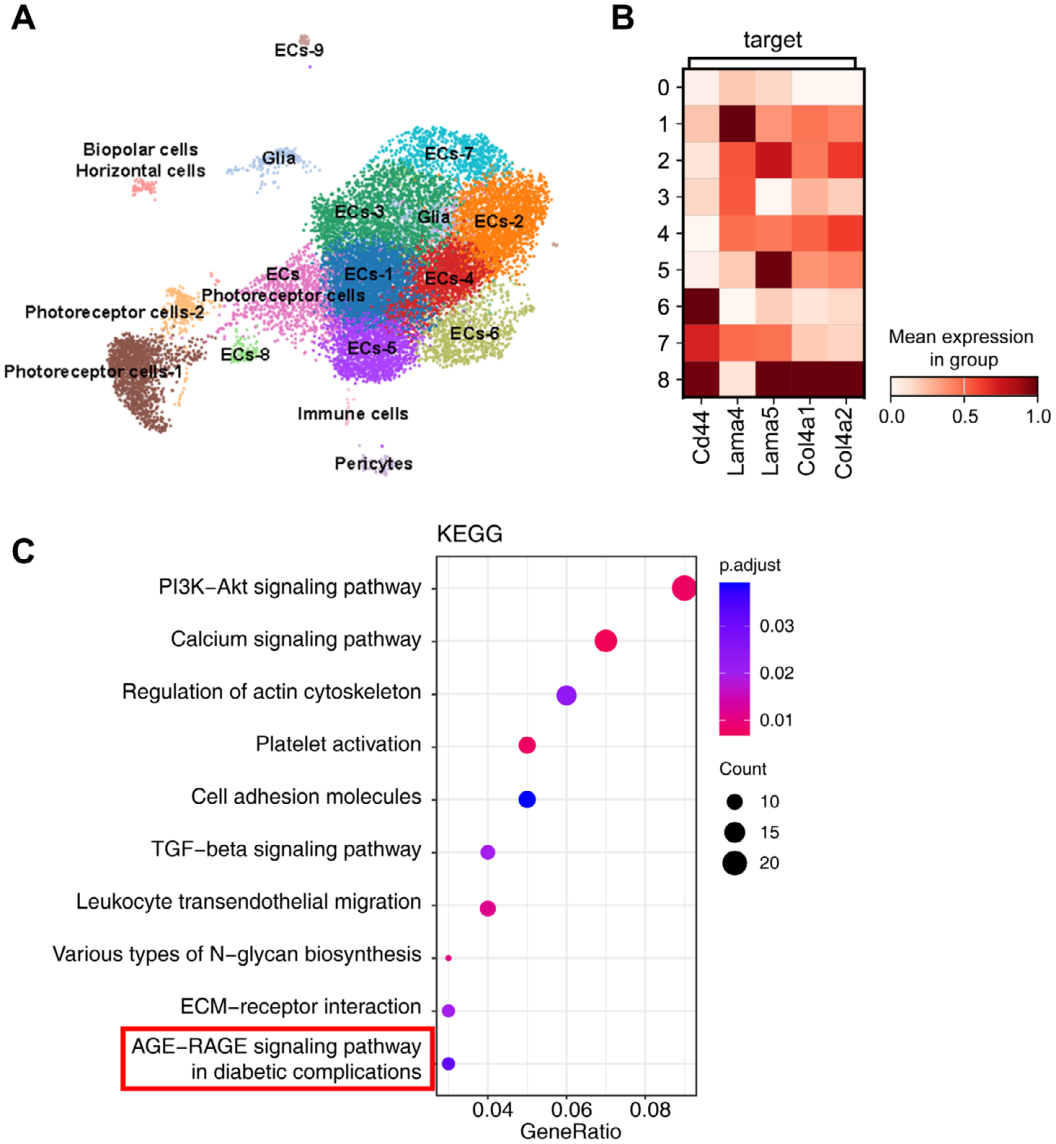
Endothelial cells exhibit enrichment of AGE‐RAGE signalling and basement membrane components during retinal vascularisation in oxygen‐induced retinopathy (OIR) mice. (A) UMAP plot depicting retinal cell clustering in OIR mice. (B) Expression of collagen IV (Col‐IV) and laminin (LN), major basement membrane components, within endothelial cell populations. (C) KEGG pathway enrichment analysis of differentially expressed genes (DEGs) in endothelial cells.

To investigate the mechanisms underlying the incomplete maturation of pathological angiogenesis in DR, we analysed scRNA‐seq data from retinal tissue of OIR mice. Through cluster analysis (Figure 1A) and cell annotation (Figure S1A), retinal cells were categorised into 16 clusters, with nine clusters identified as endothelial cells. To explore potential abnormalities in the BM during pathological angiogenesis, we examined the expression of BM components Col‐IV and LN within the endothelial cell clusters. The results demonstrated a significant upregulation of Col4a1, Col4a2 and Lama5, predominantly in endothelial cell Cluster 8, while Lama4 was enriched in endothelial cell cluster 1 (Figure 1B), although its overall expression did not differ. Further KEGG pathway analysis and GO enrichment of characteristic genes in endothelial cell clusters revealed that pathological angiogenesis induced by OIR enriched genes associated with the AGE‐RAGE signalling pathway in diabetic complications (Figure 1C). GO enrichment also concentrated on components and biological processes related to cell‐extracellular matrix/BM interactions (Figure S1B). We hypothesise that the imbalanced expression of Col‐IV and LN in different endothelial cell types, along with the enrichment of AGE‐RAGE signalling and BM‐related pathways, may contribute to the structural disarray of the vascular BM.

**FIGURE 2:**
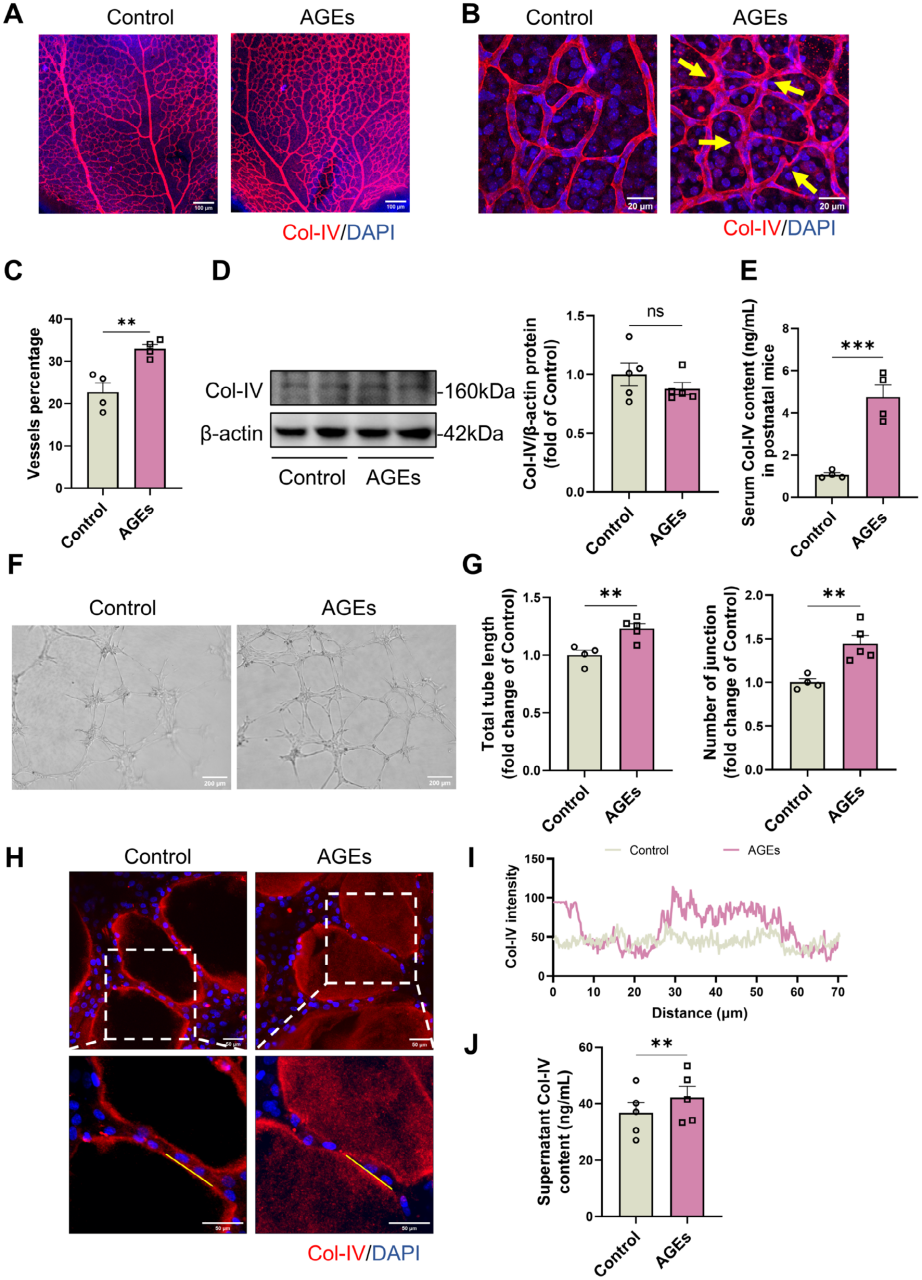
AGEs promote angiogenesis but lead to disturbed distribution of basement membrane Col‐IV. P1‐P7 wild‐type mice were injected intragastrically with 10 mg/kg of AGEs per day, and the control group was given an equal amount of BSA for 7 days. (A, B) Col‐IV immunofluorescence staining of retinal blood vessels in AGEs‐treated mice at low magnification with a scale bar of 100 μm (A) and at high magnification with a scale bar of 20 μm (B), with yellow arrows indicating abnormal thickening or loss of basement membranes. (C) retinal blood vessel density of mice was analysed by AngioTool software. *n* = 4, **p < 0.01. (D) Protein content and quantification of Col‐IV in the retinal tissues of mice treated with AGEs. *n* = 5, ns. (E) The content of Col‐IV in the serum of mice. *n* = 4, ***p < 0.001. (F) Tube formation of AGEs‐treated endothelial cells co‐cultured with pericytes with a scale bar of 200 μm and (G) total length of tubes and number of branching points analysed by AngioTool. *n* = 4–5, **p < 0.01. (H) Representative images of Col‐IV immunofluorescence staining of co‐cultured tube assay. Scale bar is 50 μm. (I) Quantitative analysis of Col‐IV distribution in a defined region (yellow line) of the image by ImageJ. (J) Determination of protein content of Col‐IV in the co‐culture supernatant. *n* = 5, **p < 0.01. All results were analysed by two‐tailed unpaired *t*‐test.

To investigate the impact of AGEs accumulation on retinal pathological angiogenesis and its effect on the BM, we conducted experiments using AGEs‐treated P1‐P7 neonatal mice and 7–8‐week‐old adult C57BL/6J mice. Immunofluorescence staining revealed that, compared to the control group, AGEs promoted angiogenesis in the retinas of neonatal mice, leading to increased vessel density (Figure 2A,C). However, Col‐IV distribution was uneven, and the BM became rough or even collapsed (Figure 2B). In adult mice, the long‐term effects of AGEs in vivo also promoted an increase in retinal vessels (Figure S2A), with uneven distribution of Col‐IV and a loss of BM smoothness (Figure S2A,B).

To elucidate the molecular mechanisms underlying BM disorganisation in pathological angiogenesis, this study analysed the other retinal RNA transcriptome sequencing data from OIR mouse model, as previously published by Binet et al. [21]. GSEA revealed that the ‘basement membrane organization’ gene set was markedly upregulated in OIR‐induced angiogenesis (Figure S3A), a finding consistent with prior scRNA‐seq results. Further differential expression analysis of BM assembly‐related genes between normoxic and OIR groups demonstrated upregulated expression of LN isoforms (Lama4 and Lama5) (Figure S3B), whereas no significant change was observed in the major collagen components of BM, Col‐IV (Col4a1 and Col4a2) (Figure S3C). This imbalanced expression of Col‐IV and LN may contribute to structural abnormalities in the vascular BM. Subsequently, we measured the levels of Col‐IV in retinal tissue and serum. The results indicated that AGEs did not affect the Col‐IV content in the retinal tissue of neonatal mice (Figure 2D), but its serum content was significantly increased (Figure 2E), suggesting potential over‐degradation. Adult mice also exhibited similar results under long‐term AGEs exposure (Figure S2C,D). In vitro, we pretreated endothelial cells with 100 μg/mL AGEs and co‐cultured them with pericytes for tube formation assays. The results demonstrated that AGEs treatment of endothelial cells promoted increased tube formation (Figure 2F,G). Immunofluorescence staining showed that the BM collagen in the tubes formed by AGEs‐treated endothelial cells was unevenly distributed (Figure 2H,I), with some areas even lacking (Figure 2H), and the Col‐IV content in the co‐culture supernatant was significantly elevated (Figure 2J). These findings suggest that AGEs can promote angiogenesis both in vivo and in vitro, while also disrupting the structure of the BM.

### 3.2 AGEs Promote Angiogenesis via the Upregulation of CD44 Expression in Endothelial Cells, Leading to Alterations in BM Architecture

**FIGURE 3:**
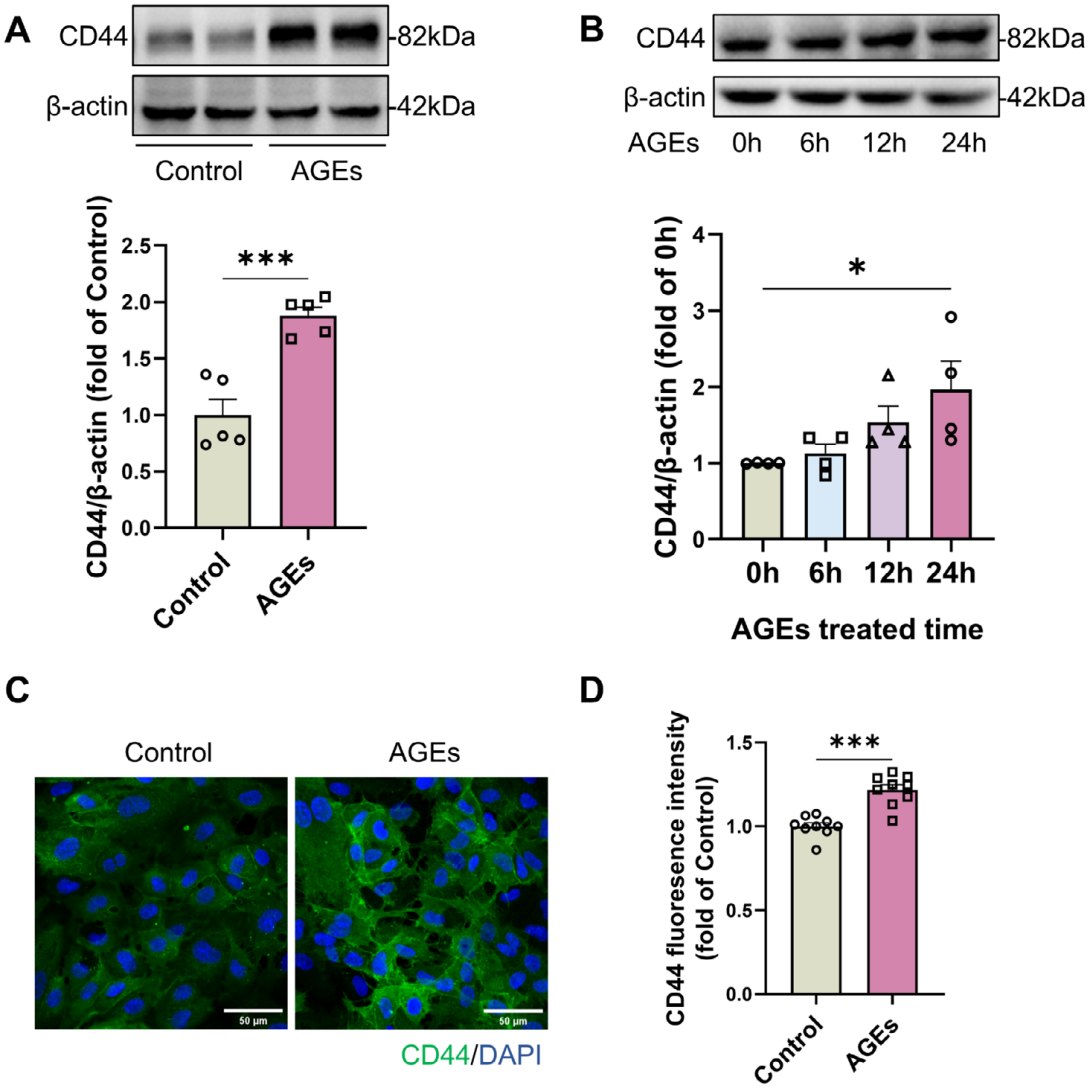
AGEs promote CD44 protein expression in vitro and in vivo. (A) Expression and quantitative analysis of CD44 protein in retina of AGEs‐treated suckling mice. Statistical analysis was performed using two‐tailed unpaired *t*‐test; *n* = 5, ***p < 0.001. (B) Expression of CD44 protein in AGEs‐treated HUVECs. The results were statistically analysed by one‐way ANOVA and Dunnett's test. *n* = 4, *p < 0.05. (C, D) CD44 immunofluorescence staining in HUVECs treated with AGEs. Quantification was performed using ImageJ. The results were analysed by two‐tailed unpaired *t*‐test; *n* = 5, ***p < 0.001.

To explore the effect of CD44 in AGEs‐induced disruption of the neovascular BM structure, we examined CD44 protein expression in AGEs‐treated mouse retinal tissues. Western blot analysis revealed a significant increase in CD44 protein levels in the retinas of neonatal mice following AGEs treatment compared to the control group (Figure 3A). CD44 levels were also elevated in the retinal tissues of adult mice, with a statistically significant increase observed after 6 months of AGEs exposure (Figure S4A,B). In vitro, HUVECs were treated with 100 μg/mL AGEs, and total cellular protein was extracted after 24 h. The results demonstrated that AGEs promoted CD44 expression in endothelial cells in a time‐dependent manner (Figure 3B). Immunofluorescence staining revealed that, compared to the control group, CD44 clustered on the endothelial cell membrane after AGEs treatment (Figure 3C), with a significant increase in overall fluorescence intensity (Figure 3D). These findings indicate that AGEs can promote CD44 protein expression in endothelial cells in vitro.

**FIGURE 4:**
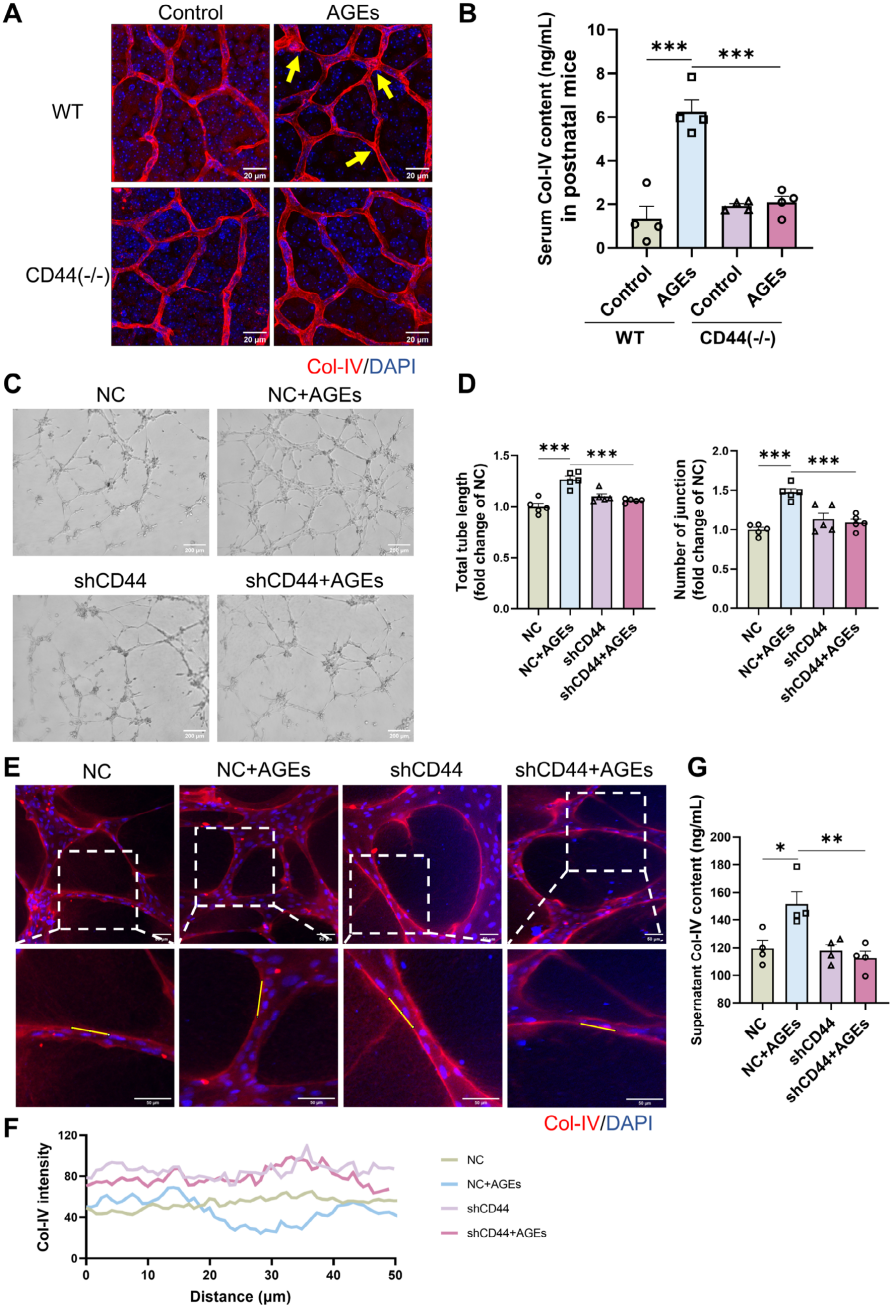
CD44 is involved in AGEs‐induced angiogenesis and vascular basement membrane disorders. (A) Retinal Col‐IV fluorescence staining of AGEs‐treated WT and CD44 knockout mice, showing representative images from four independent experiments, with yellow arrows indicating abnormal thickening of the basement membrane. (B) serum Col‐IV levels of AGEs‐treated WT and CD44 knockout mice, analysed statistically using one‐way ANOVA and Tukey's multiple comparison test; *n* = 4, ***p < 0.001. (C) Representative image of tube formation in co‐culture of pericytes with HUVECs of down‐regulated CD44 expression. (D) Total vessel length and number of branching nodes of forming tubes in HUVECs‐pericytes co‐culture were analysed by AngioTool software and statistically analysed using one‐way ANOVA and Tukey's multiple comparison test; *n* = 5, ***p < 0.001. (E) Representative images of Col‐IV immunofluorescence staining of vascular basement membranes formed in HUVECs‐pericytes co‐culture. (F) Quantitative analysis of Col‐IV distribution in a defined region (yellow line) of the image by ImageJ. (G) Col‐IV content in co‐culture supernatants, and statistically analysed by one‐way ANOVA and Tukey's multiple comparison test; *n* = 4, *p < 0.05, **p < 0.01.

To investigate the impact of CD44 on AGEs‐induced pathological changes in the vascular BM, we conducted experiments using CD44 gene knockout mice (Figure S5A). Initially, we validated the CD44 knockout efficiency at both the gene and protein levels in tissues (Figure S5B,C). Subsequently, we treated the neonatal mice with AGEs and, after 7 days, harvested retinal tissues and serum for analysis. Retinal Col‐IV staining revealed that AGEs treatment led to increased vascularisation in the neonatal mouse retina, accompanied by uneven collagen distribution. However, in CD44 knockout mice, the AGEs‐induced neovascularisation and BM collagen disarray were attenuated (Figure 4A). The AGEs‐induced increase in Col‐IV levels in serum was also reversed in CD44 knockout mice (Figure 4B). The changes in the major non‐collagenous BM component, LN, in retinal tissues and serum (Figure S6A,B) mirrored the Col‐IV findings. Furthermore, we knocked down CD44 expression in HUVECs in vitro (Figure S5D,E) and subsequently treated the cells with AGEs. After 24 h, we co‐cultured the HUVECs with HBVPs for a tube formation assay. The results demonstrated that downregulating CD44 protein expression in HUVECs mitigated the AGEs‐induced increase in tube formation in the endothelial‐pericyte co‐culture (Figure 4C,D). Immunofluorescence staining of the formed vessels for Col‐IV and LN revealed that the downregulation of CD44 in endothelial cells partially ameliorated the structural disorganisation of the neovascular BM (Figure 4E,F, Figure S6C), and the levels of Col‐IV (Figure 4G) and LN (Figure S6D) in the co‐culture supernatant were also reduced. These findings suggest that increased CD44 expression in endothelial cells plays a crucial role in AGEs‐induced neovascularisation and BM abnormalities.

### 3.3 MMP9‐Mediated BM Degradation Contributes to the Structural Abnormalities of the Neovascular BM Induced by AGEs

**FIGURE 5:**
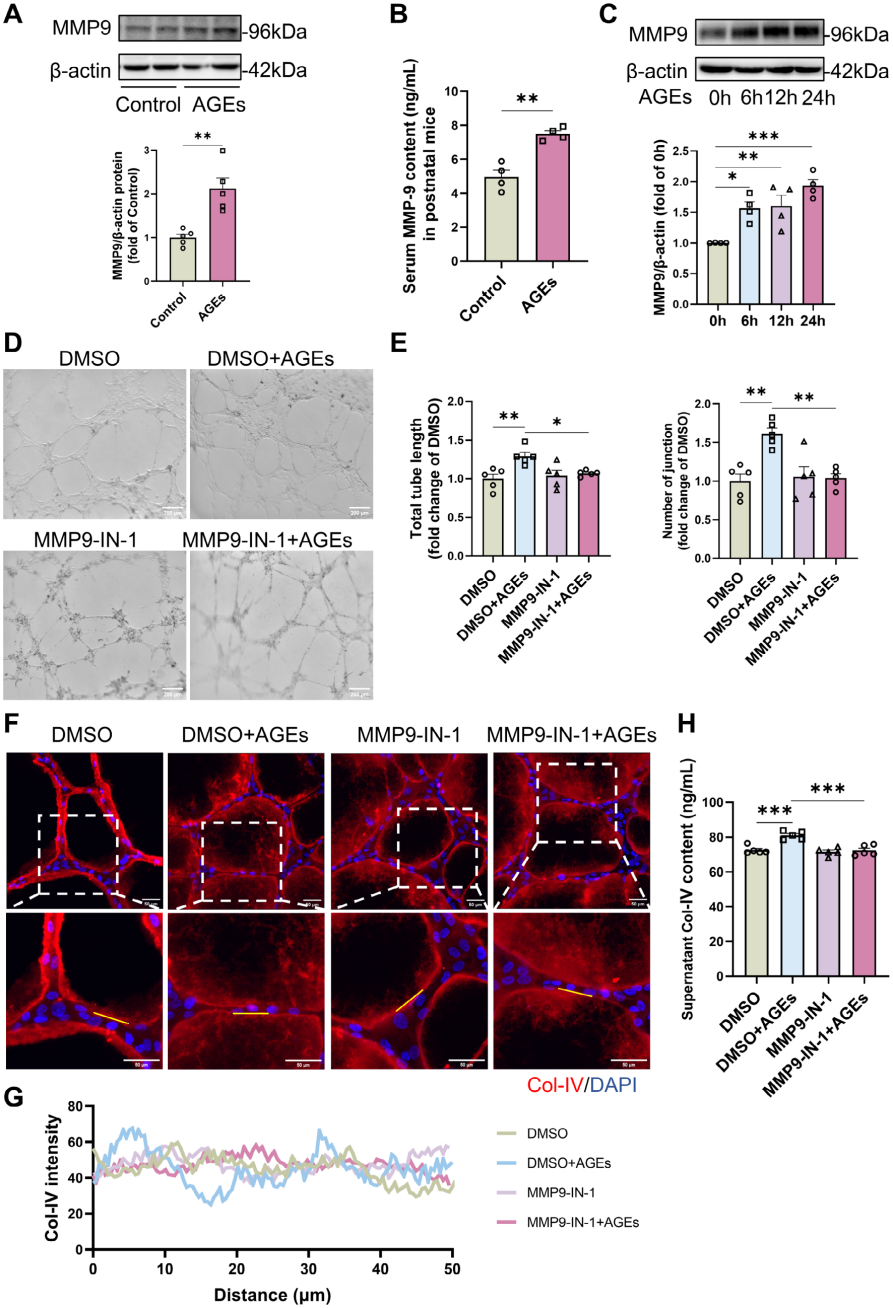
AGEs degrade Col‐IV by promoting MMP9 expression leading to structural disorganisation of basement membrane. (A) Protein content and quantification of MMP9 in retinal tissues of newborn suckling mice treated with 10 mg/kg AGEs for 7 days. Results were analysed by two‐tailed unpaired *t*‐test, *n* = 5, **p < 0.01. (B) MMP9 secretion in serum of suckling mice treated with AGEs. Results were statistically analysed by two‐tailed unpaired *t*‐test, *n* = 4, **p < 0.01. (C) Protein expression and quantitative analysis of MMP9 after treatment of HUVECs with AGEs. One‐way ANOVA and Dunnett's test were used for statistical analysis, *n* = 4, *p < 0.05, **p < 0.01, ***p < 0.001. (D, E) Representative image of tube formation and quantitative analysis in co‐culture of MMP9‐IN‐1‐treated HUVECs with pericytes. Scale bar is 200 μm. Results were analysed by one‐way ANOVA and Tukey's test, *n* = 5, **p < 0.01, *p < 0.05. (F) Representative plots of Col‐IV fluorescence staining in tubes of co‐culture of MMP9‐IN‐1‐treated HUVECs with pericytes on a 50 μm scale. (G) Quantitative analysis of Col‐IV distribution in a defined region (yellow line) in tubes co‐cultured with MMP9‐IN‐1‐treated HUVECs. (H) Col‐IV content in supernatants from co‐culture of MMP9‐IN‐1‐treated HUVECs with pericytes. Results were analysed by one‐way ANOVA and Tukey's test, *n* = 5, ***p < 0.001.

The matrix metalloproteinase (MMP) family of proteins is the primary proteinases responsible for the degradation of BM components, with MMP9 being the primary enzyme responsible for the degradation of Col‐IV. Consequently, we assessed the expression and secretion of MMP9 in both AGEs‐treated mice and endothelial cells. Western blot analysis revealed that AGEs promoted the expression of MMP9 in the retinas of neonatal mice (Figure 5A), accompanied by a significant increase in the levels of MMP9 secreted into the serum (Figure 5B). In HUVECs, AGEs similarly enhanced the protein expression of MMP9 (Figure 5C). To investigate the relationship between endothelial cell MMP9 and the degradation of neovascular BM, we employed the specific MMP9 inhibitor, MMP9‐IN‐1, which selectively targets the heme domain of MMP9 without affecting other MMPs, to treat HUVECs (Figure S7A) before AGEs stimulation. Then HUVECs were collected and co‐cultured with HBVPs. The results demonstrated that MMP9‐IN‐1 treatment in endothelial cells reduced AGEs‐induced angiogenesis (Figure 5D,E), suggesting its involvement in the neovascularisation process. Immunofluorescence staining of Col‐IV and LN revealed that pretreatment with MMP9‐IN‐1 mitigated the AGEs‐induced disruption of neovascular BM structure, restoring the continuous and smooth distribution of Col‐IV (Figure 5F,G) and LN (Figure S7B). Pretreatment with MMP9‐IN‐1 in HUVECs also attenuated the increased levels of AGEs‐induced Col‐IV (Figure 5H) and LN (Figure S7C) in co‐culture supernatant, indicating decreased degradation. These findings suggest that MMP9 mediates AGEs‐induced angiogenesis and the disruption of BM structure through excessive degradation of the BM.

### 3.4 AGEs Promote Endothelial MMP9 Expression via CD44, Leading to the Degradation of the Vascular BM

**FIGURE 6:**
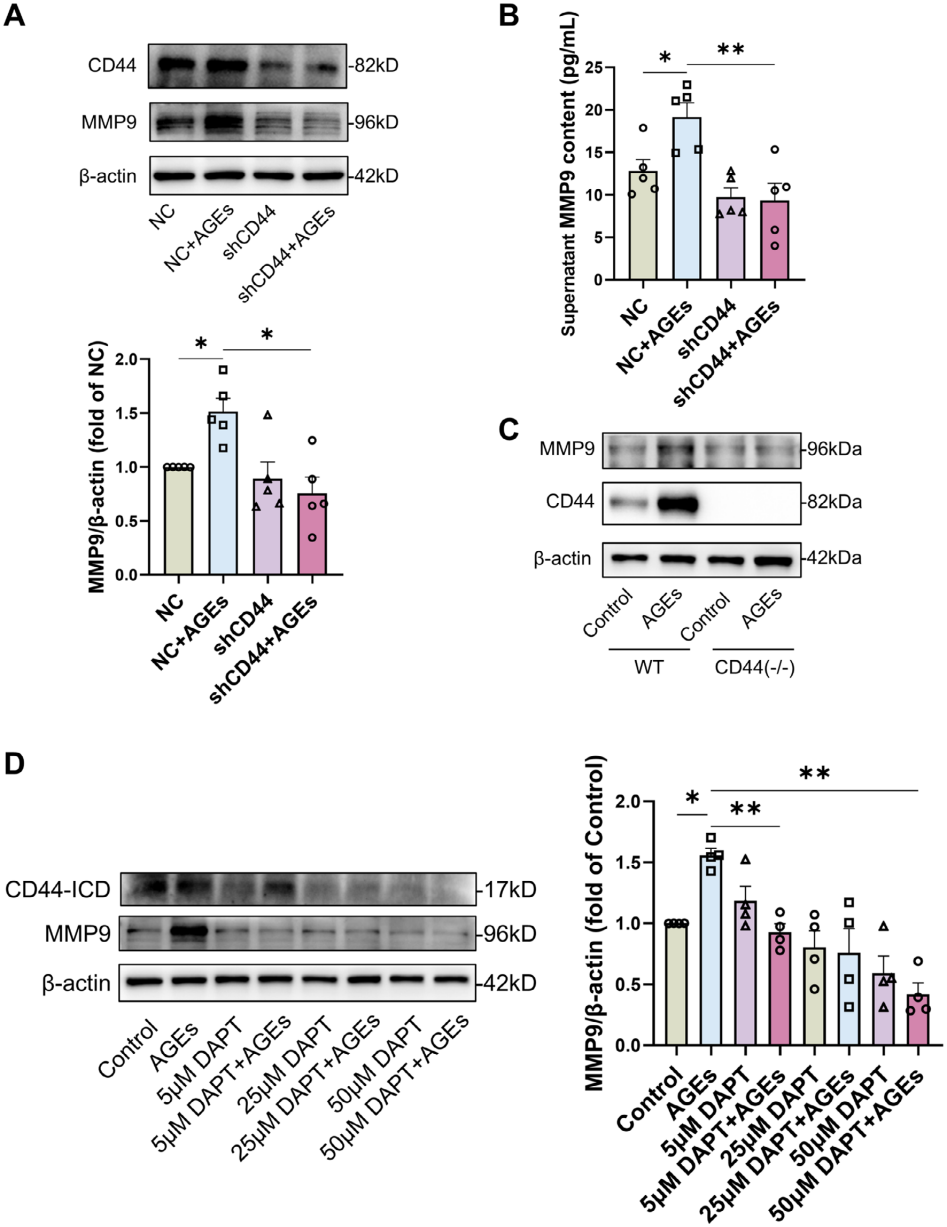
AGEs promote MMP9 expression via CD44, thereby leading to structural disruption of the neovascular basement membrane. (A) Protein expression and quantitative analyses of MMP9 in HUVECs transfected with adenovirus shCD44. Results were analysed by one‐way ANOVA and Tamhane's test, *n* = 5, *p < 0.05. (B) MMP9 content in supernatants from co‐culture of shCD44‐transfected HUVECs with pericytes. Results were analysed by one‐way ANOVA and Tukey's test, *n* = 5, *p < 0.05, **p < 0.01. (C) MMP9 protein content in retinal tissues of AGEs‐treated CD44‐knockout suckling mice after 7 days of AGEs treatment; (D) CD44ICD fragment formation and MMP9 protein expression in HUVECs treated with DAPT at different concentrations. Quantitative analyses of MMP9 were analysed by one‐way ANOVA and Tamhane's test, *n* = 4, *p < 0.05, **p < 0.01.

Previous research has indicated that CD44 undergoes cleavage to generate intracellular fragments, thereby promoting the expression of genes including MMP9 [28]. We employed shCD44 to downregulate CD44 expression in endothelial cells, followed by AGEs treatment. The results demonstrated that CD44 downregulation significantly reduced AGEs‐induced MMP9 protein expression (Figure 6A). Co‐culturing shCD44‐treated endothelial cells with pericytes resulted in decreased MMP9 levels in the supernatant (Figure 6B). CD44 knockout mice presented a reduction of AGE‐induced MMP9 protein expression (Figure 6C), further supporting the role of CD44 in promoting MMP9 expression. To investigate whether AGEs accumulation in diabetes also induces CD44 cleavage and the formation of CD44 intracellular domain (ICD) fragments, and whether CD44ICD is associated with MMP9 expression, we utilised the γ‐secretase inhibitor DAPT to inhibit CD44ICD fragment formation (Figure 6D). We observed a significant downregulation of AGEs‐induced MMP9 protein expression (Figure 6D), indicating that CD44 cleavage and the formation of ICD fragments promote AGEs‐induced MMP9 expression.

### 3.5 AGEs Augment CD44 Expression via Β‐Catenin/TCF4 Signalling Pathway, Thereby Mediating Aberrant Neovascular BM Homeostasis

**FIGURE 7:**
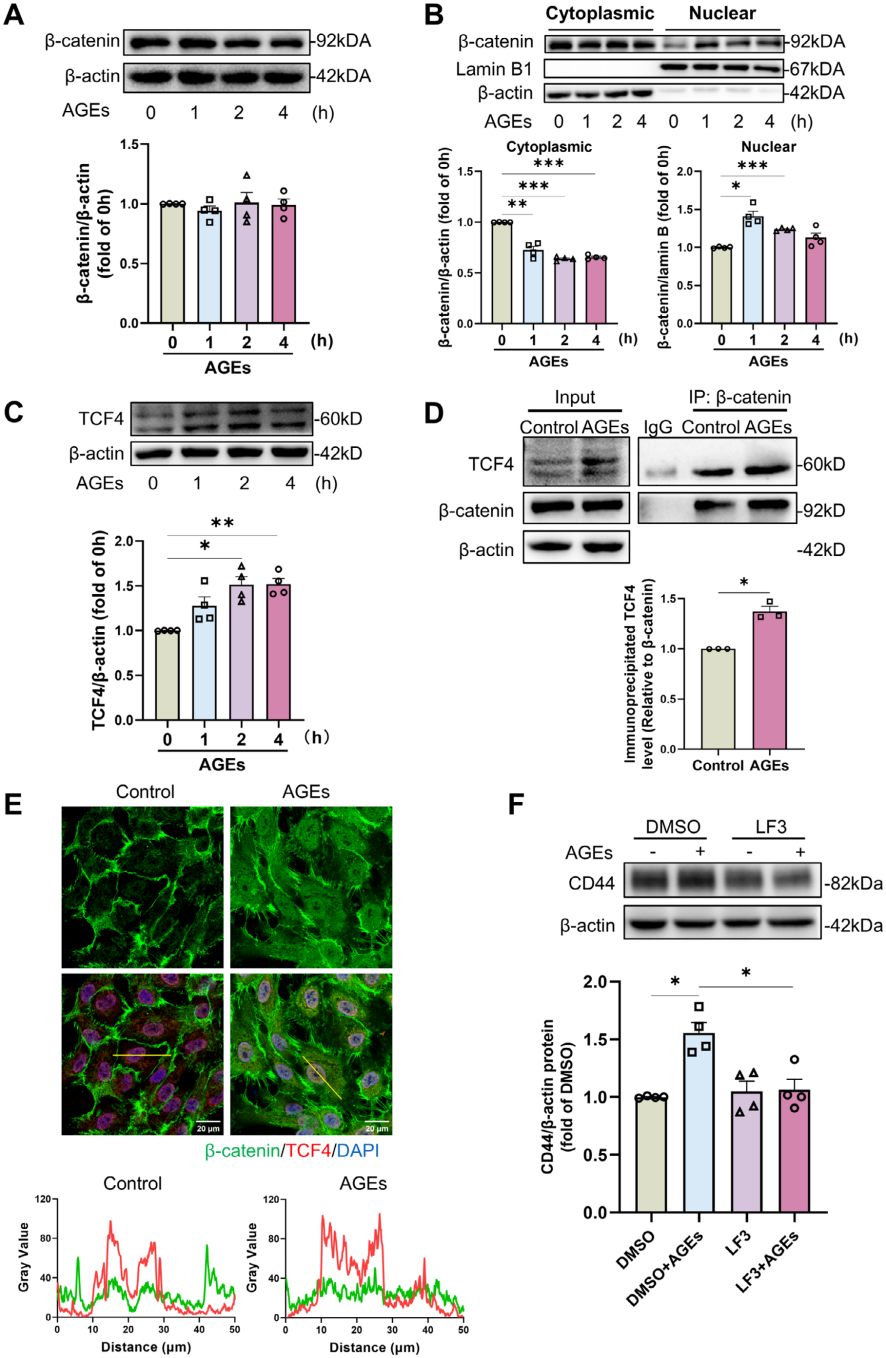
AGEs increase endothelial cell CD44 expression through the β‐catenin/TCF4 signalling pathway. (A) Total protein expression of β‐catenin in AGEs‐treated HUVECs, and (B) Nuclear and cytoplasmic protein levels of β‐catenin in HUVECs treated with AGEs. One‐way ANOVA and Dunnett's test were used for statistical analysis; *n* = 4, *p < 0.05, **p < 0.01, ***p < 0.001. (C) Protein expression of TCF4 in AGEs‐treated HUVECs. The results were analysed by one‐way ANOVA and Dunnett's test, *n* = 4, *p < 0.05, **p < 0.01. (D) The interaction of β‐catenin with TCF4 detected with Co‐IP. Results were analysed by two‐tailed unpaired *t*‐test, *n* = 3, *p < 0.05. (E) Immunofluorescence image and fluorescence distribution of β‐catenin and TCF4 in HUVECs treated with AGEs. (F) Protein expression and quantitative analyses of CD44 in LF3‐treated HUVECs. Statistical analysis was performed using one‐way ANOVA and Tamhane's test, *n* = 4, *p < 0.05.

Extensive research has demonstrated that CD44 serves as a canonical target gene within the Wnt/β‐catenin signalling pathway. To investigate whether AGEs induce increased endothelial CD44 expression via the β‐catenin signalling pathway, we assessed the total and subcellular levels of β‐catenin in HUVECs following AGEs treatment. Our findings revealed that AGEs did not alter the total β‐catenin protein levels within a short timeframe (Figure 7A), but facilitated its translocation into the nucleus (Figure 7B). Concurrently, the total protein levels of TCF4 were elevated in AGEs‐treated endothelial cells (Figure 7C). The results of the co‐immunoprecipitation experiments indicate that AGEs can enhance the interaction between β‐catenin and TCF4 both in vitro and in vivo (Figure 7D, Figure S8A). Immunofluorescence staining of endothelial cells further confirmed the enhanced nuclear co‐localisation of β‐catenin and TCF4 (Figure 7E). To determine the involvement of the β‐catenin/TCF4 pathway in AGEs‐induced CD44 expression, we employed LF3, a specific antagonist that inhibits β‐catenin/TCF4 interaction (Figure S8B). Pretreatment of HUVECs with LF3, followed by AGEs exposure, demonstrated that the LF3‐mediated inhibition of β‐catenin/TCF4 interaction significantly attenuated the AGEs‐induced increase in CD44 protein levels (Figure 7F), indicating that AGEs promote CD44 expression in endothelial cells through this pathway.

Furthermore, we investigated the role of the β‐catenin/TCF4 pathway in AGEs‐induced angiogenesis. Following LF3 treatment of endothelial cells, we co‐cultured them with pericytes to assess tube formation. The results demonstrated that LF3 significantly mitigated AGEs‐induced vessel formation (Figure S8C,D) and the uneven distribution of collagen (Figure S8E,F), indicating the involvement of the β‐catenin/TCF4 pathway in AGEs‐mediated angiogenesis and BM abnormalities. These findings suggest that AGEs promote increased CD44 expression in endothelial cells via the β‐catenin/TCF4 signalling pathway, thereby mediating angiogenesis and disrupting the BM structure.

## 4 Discussion

This study systematically elucidates the molecular mechanisms by which AGEs induce pathological angiogenesis and BM abnormalities via the CD44‐MMP9 axis. The results further clarify the role of the β‐catenin/TCF4 signalling pathway in this process. Prior research on CD44 has primarily focused on its roles in inflammation and cell migration. Recent studies have implicated CD44 in metabolic diseases such as obesity and diabetes through metabolic regulation and the mediation of insulin resistance [29, 30, 31]. This study investigates the relationship between CD44 and the construction of neovascular BMs, proposing for the first time that CD44 serves as a central molecule linking AGEs signalling to BM remodelling, thereby revealing a novel function in BM homeostasis. AGEs activate the β‐catenin/TCF4 pathway, significantly upregulating CD44 expression in endothelial cells. Subsequent activation of CD44 promotes the synthesis and secretion of MMP9, leading to the excessive degradation of BM components, including Col‐IV and LN. Notably, CD44ICD participates in the transcriptional regulation of MMP9 through γ‐secretase‐dependent cleavage. This mechanism offers potential therapeutic strategies by targeting the CD44 cleavage process, potentially minimising interference with other physiological functions of CD44. Furthermore, in contrast to the conventional understanding that AGEs disrupt the BM solely through direct collagen cross‐linking, this study proposes a novel mechanism by which AGEs indirectly induce vascular BM disruption through ‘synthesis‐degradation imbalance’. Although the overall expression of Col‐IV remains unchanged, its uneven distribution and elevated serum levels suggest enhanced local degradation, providing a new framework for understanding vascular leakage and haemorrhage in the development of proliferative DR.

CD44 promotes tumour invasion and metastasis by facilitating pathological angiogenesis [32]. In a study on myocardial infarction, the expression of CD44 in the border zone of the infarct is closely associated with pathological angiogenesis. The absence of CD44 impairs angiogenesis and affects the biosynthesis and pro‐angiogenic function of plasma exosomes [33]. By using CD44 knockout mice and adenovirus‐mediated interference with endothelial CD44 expression, our study demonstrates that CD44 deficiency alleviates angiogenesis induced by the accumulation of AGEs. Although anti‐angiogenic therapy is the mainstream strategy for treating pathological angiogenesis diseases such as proliferative DR and can effectively reduce blood vessel formation, it only transforms the disease from a lethal stage to a chronic asymptomatic state, which is prone to recurrence. Moreover, prolonged and repeated use of anti‐angiogenic drugs can lead to drug resistance, significantly diminishing therapeutic efficacy. Based on anti‐angiogenic theory, Jain proposed the concept of ‘vascular normalisation’ to address the limitations of anti‐angiogenic therapy [34]. Vessel normalisation therapy has shown benefits not only in preclinical trials but also in clinical studies. Here we reveal that modulating endothelial CD44 expression in vivo and in vitro could mitigate AGEs‐induced uneven distribution of Col‐IV and LN in vascular BM, indicating that CD44 mediates BM structural abnormalities in pathological angiogenesis. Studies have shown that CD44 can promote the activation of MMP9 [35, 36, 37]. After intervening in CD44 expression or using a γ‐secretase inhibitor to suppress CD44ICD formation, we observed a reduction in MMP9 protein levels, suggesting that CD44 is also closely associated with MMP9 expression. Furthermore, our previous research demonstrated that CD44 could cluster with phosphorylated moesin in pericytes, affecting endothelial cell‐pericyte interactions and leading to inadequate neovascular maturation [17, 19]. These results highlight the significant potential of CD44 in vascular normalisation therapy.

However, this study also has certain limitations. Firstly, while the research focused on the endothelial cell‐CD44 pathway, the involvement of pericytes or other retinal cells, such as glial cells, in BM remodelling via paracrine signalling remains unclear. Secondly, the conclusions are primarily based on an AGEs‐treated angiogenesis animal model and in vitro co‐culture experiments. Synchronous validation in traditional diabetic mouse models is lacking, and the clinical relevance of the CD44‐MMP9 axis has not been verified in tissue samples from patients with DR.

Despite the aforementioned limitations, the signalling axis elucidated in this study offers a novel and potentially translational approach for therapeutic intervention in related diseases. Its clinical translational significance lies in identifying multiple molecular targets for vascular complications such as DR, including CD44, MMP9 and upstream signalling regulators. These insights facilitate the development of targeted therapeutics, notably CD44 antagonists, to stabilise the BM and promote vascular maturation. Extensive evidence supports the efficacy, at least in animal models, of CD44‐targeted anti‐oncogenic strategies—including monoclonal antibodies, peptides, aptamers, small‐molecule inhibitors, as well as gene and cell therapies and biomaterials [38]. A study demonstrated that hyaluronan nanoparticle treatment in high‐fat diet‐induced murine models, which disrupts HA‐CD44 interactions, can improve insulin sensitivity [39]. Our data further indicate that CD44 knockout alleviates vascular BM abnormalities associated with pathological neovascularisation in diabetes. Consequently, applying anti‐CD44 therapies for type 2 diabetes presents a feasible and promising avenue. Additionally, this study suggests that serum levels of BM components like Col‐IV or soluble MMP9 could serve as potential diagnostic and prognostic biomarkers, providing a theoretical foundation for therapies aimed at promoting vascular normalisation rather than solely inhibiting angiogenesis.

In summary, this study elucidates the mechanism by which AGEs disrupt BM architecture and promote pathological angiogenesis through the β‐catenin/TCF4‐CD44‐MMP9 signalling axis, thereby enhancing the understanding of the pathogenesis of diabetic vascular complications and establishing a vital molecular foundation for the development of innovative therapies aimed at vascular normalisation.

## 5 Conclusion

This study elucidates a novel mechanism by which AGEs disrupt the vascular BM homeostasis via the β‐catenin/CD44/MMP9 axis, thereby providing a theoretical basis for targeting CD44‐MMP9 for vascular normalisation therapy and offering a new idea for the treatment of diabetic neovascularisation.

## Author Contributions

**Xiaoxia Huang:** writing – original draft, data curation, formal analysis, investigation, validation, methodology, conceptualisation. **Zhuanhua Liu:** writing – review and editing, investigation, data curation, validation. **Jiaqing Hu:** investigation, data curation, formal analysis. **Bingyu Li:** investigation, data curation. **Zhenfeng Chen:** data curation, formal analysis. **Tairan Zeng:** data curation. **Xing Zhou:** data curation. **Ruimin Lu:** data curation. **Wenyan Deng:** data curation. **Wendong Zhou:** data curation. **Qiaobing Huang:** writing – review and editing, supervision, project administration, funding acquisition, methodology, conceptualisation.

## Funding

This work was supported by the National Natural Science Foundation of China (81370226, 81870210) and the Basic and Applied Basic Research Foundation of Guangdong Province (2023A1515010094).

## Conflicts of Interest

The authors declare no conflicts of interest.

## Supporting information


**Figure S1:** Cellular annotation and endothelial cell pathway enrichment in OIR retinal single‐cell transcriptome sequencing.
**Figure S2:** AGEs cause structural disorganisation of the vascular BM in adult mice by degrading Col‐IV.
**Figure S3:** Transcriptome Sequencing Analysis of retinal tissue from normoxic and OIR mice.
**Figure S4:** AGEs promote CD44 expression in adult mouse retinal tissues.
**Figure S5:** Knockout/knockdown of CD44 at in vivo and ex vivo levels.
**Figure S6:** CD44 knockdown alleviated AGEs‐induced maldistribution of LN in neonatal mice angiogenesis.
**Figure S7:** MMP9 inhibition affects the distribution of LN in the vascular basement membrane of HUVECs co‐cultured with pericytes.
**Figure S8:** Interaction of β‐catenin with TCF4 is involved in AGEs‐induced angiogenesis and its BM structural abnormalities.

**Table S1:** Primer sequences for genotyping of CD44(−/−) mice.
**Table S2:** List of antibodies.

## Data Availability

Data sharing not applicable to this article as no datasets were generated or analysed during the current study.
