# Supplementary material for: Increased CD44 Expression in Endothelial Cells Induced by Advanced Glycation End Products Leads to Insufficient Maturation of Angiogenesis
Source: J Cell Mol Med. 2026 Mar 16;30(6):e71088. doi: 10.1111/jcmm.71088 (PMC13097380; doi:10.1111/jcmm.71088)
Supplement: Supplementary file 1 — Figure S1: Cellular annotation and endothelial cell pathway enrichment in OIR retinal single‐cell transcriptome sequencing. Figure S2: AGEs cause structural disorganisation of the vascular BM in adult mice by degrading Col‐IV. Figure S3: Transcriptome Sequencing Analysis of retinal tissue from normoxic and OIR mice. Figure S4: AGEs promote CD44 expression in adult mouse retinal tissues. Figure S5: Knockout/knockdown of CD44 at in vivo and ex vivo levels. Figure S6: CD44 knockdown alleviated AGEs‐induced maldistribution of LN in neonatal mice angiogenesis. Figure S7: MMP9 inhibition affects the distribution of LN in the vascular basement membrane of HUVECs co‐cultured with pericytes. Figure S8: Interaction of β‐catenin with TCF4 is involved in AGEs‐induced angiogenesis and its BM structural abnormalities. [file JCMM-30-e71088-s002.docx]

**Supplementary Figures**


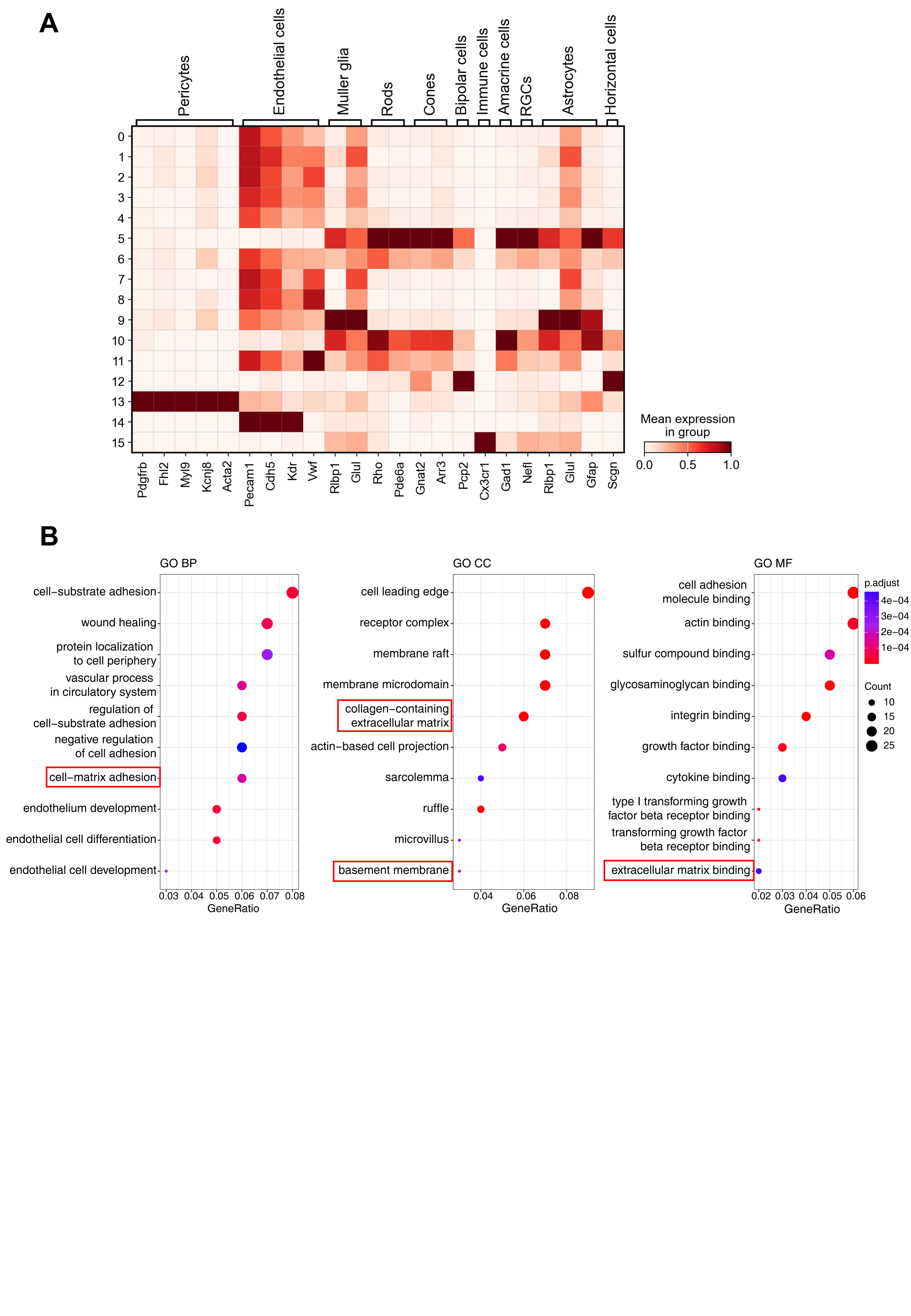


**Figure S1.** Cellular annotation and endothelial cell pathway enrichment in OIR retinal single-cell transcriptome sequencing. (A) Key marker genes for cellular annotation in OIR mouse retinal single-cell sequencing. (B) GO enrichment analysis of differentially expressed genes in OIR retinal endothelial cell clusters.


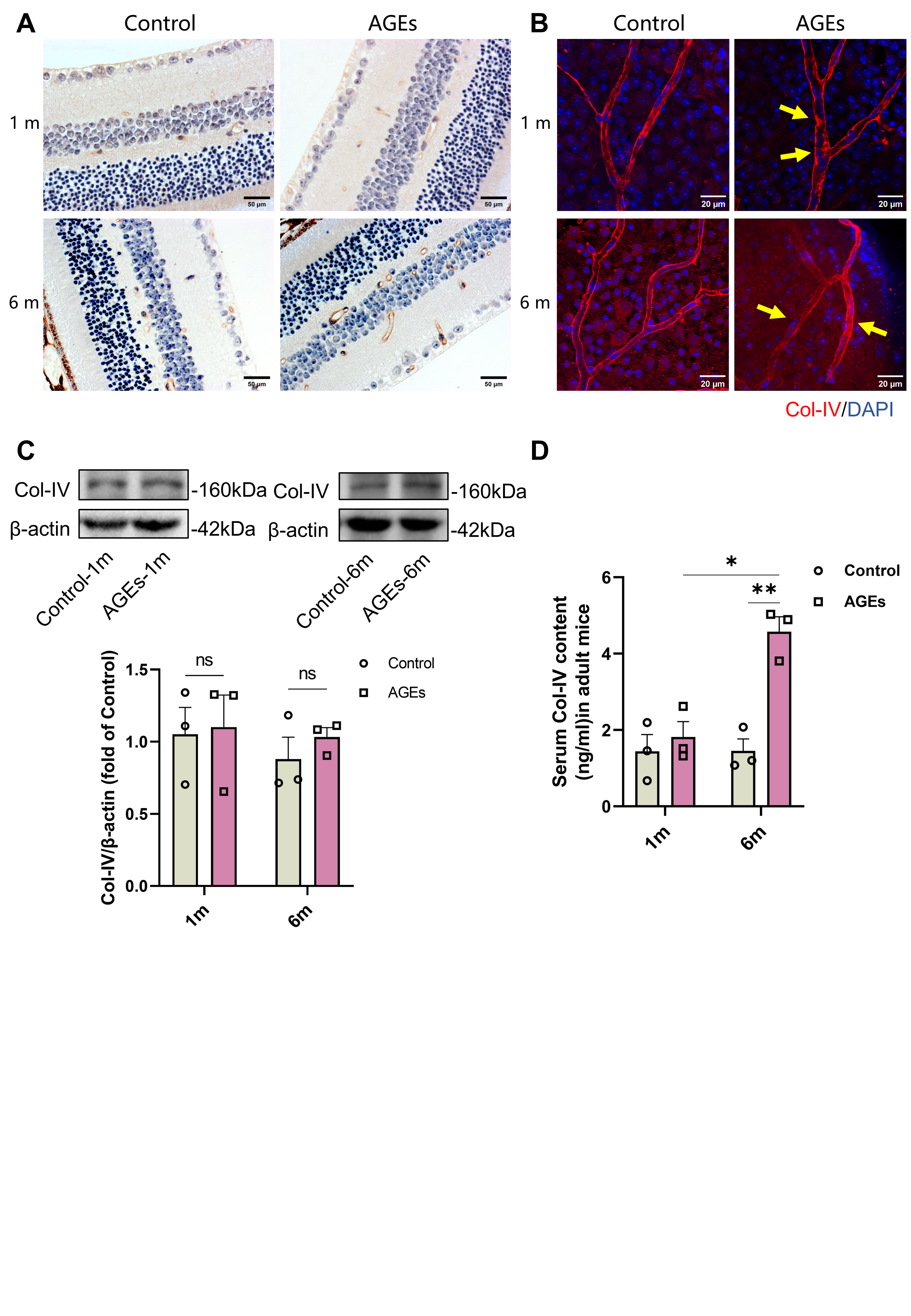


**Figure S2.** AGEs cause structural disorganization of the vascular BM in adult mice by degrading Col-IV. (A) Immunohistochemical staining of Col-IV in the retina of adult mice treated with AGEs for 1 m or 6 m on a scale of 50 μm; (B) Immunofluorescence staining of Col-IV in retinal vessels of adult mice treated with AGEs on a scale of 20 μm; (C) Protein content of Col-IV in retinal tissues of adult mice treated with AGEs and gray scale statistics, and the results were analyzed by two-way ANOVA test, n = 3, ns; (D) Protein content of Col-IV in the serum of adult mice. Results were statistically tested by two-way ANOVA, n = 3, ^*^*p* < 0.05, ^**^*p* < 0.01.


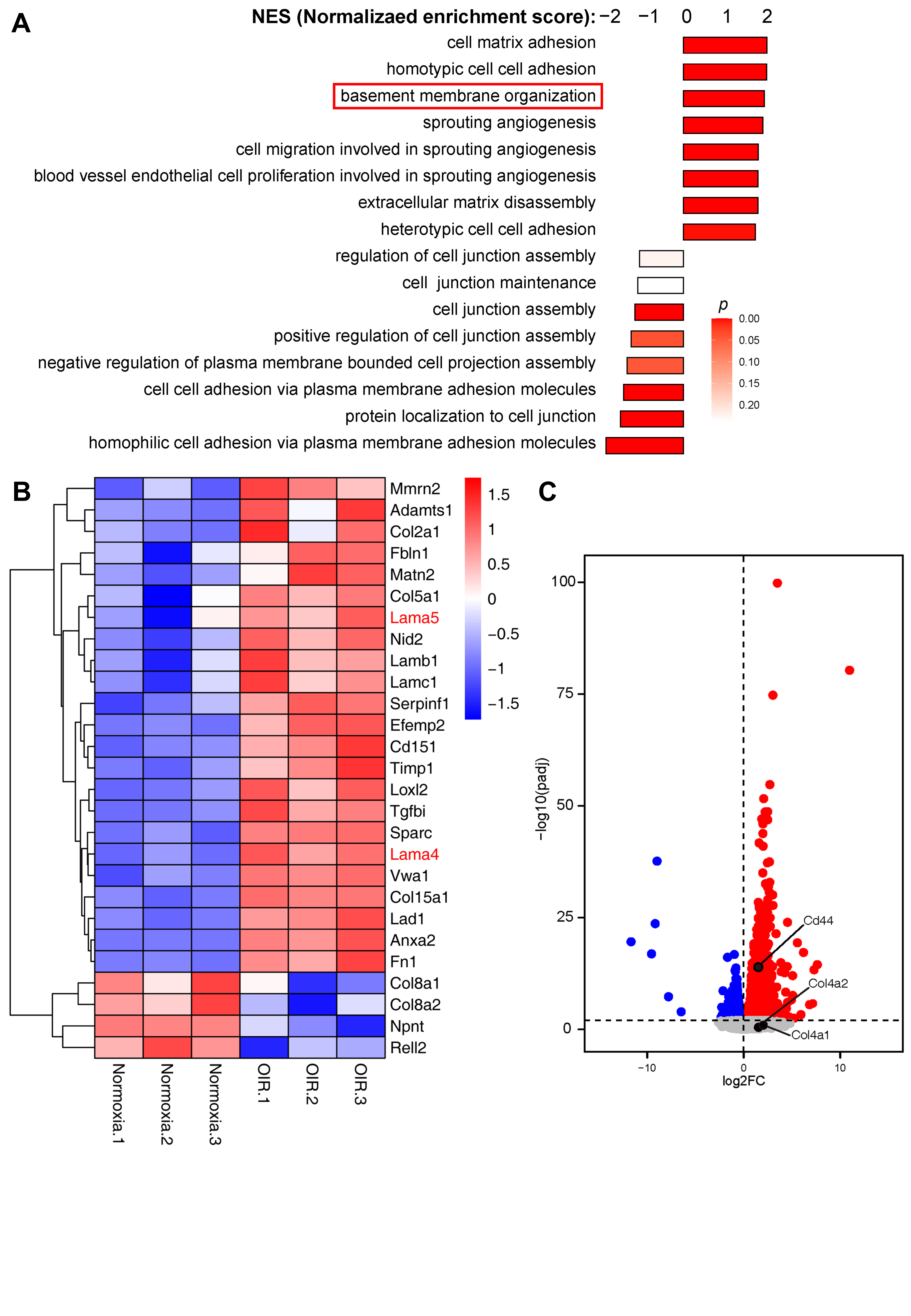


**Figure S3.** Transcriptome Sequencing Analysis of retinal tissue from normoxic and OIR mice. (A) GSEA analysis of RNA transcripts in the retinas of normoxic and OIR mice; (B) Expression of differentially expressed genes (DEGs) enriched in the "basement membrane organization" gene set in the retina of normoxia and OIR mice; (C) Compared with the normoxia group, the volcano diagram of DEGs and gene labeling in the OIR group, with red for up-regulated genes, blue for down-regulated genes, and gray for non-differentially expressed genes.


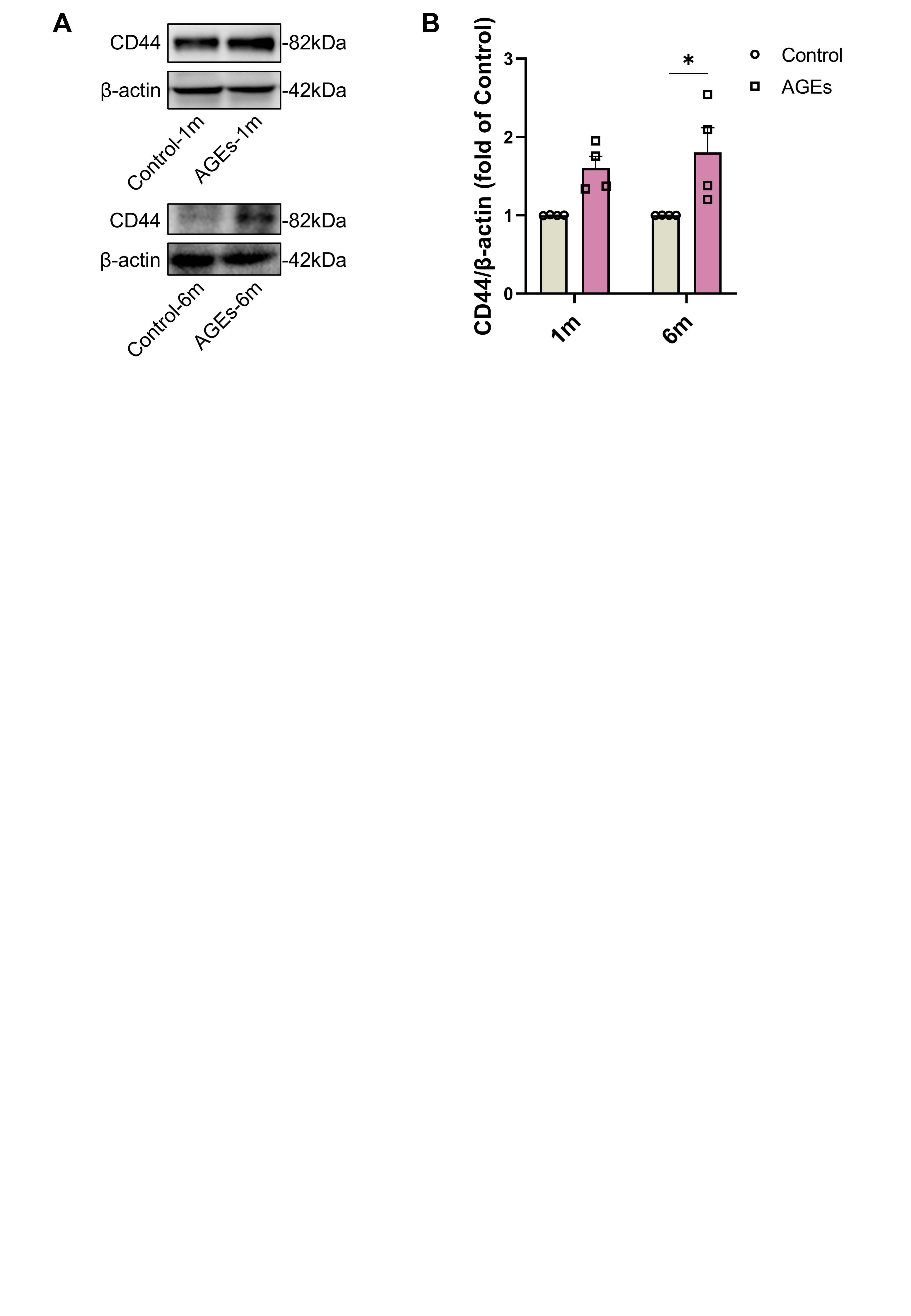


**Figure S4.** AGEs promote CD44 expression in adult mouse retinal tissues. (A) CD44 protein expression in retinal tissues of adult mice after 1 m and 6 m treatment with AGEs. (B) Statistical analysis of CD44 protein content. The results were tested by two-way ANOVA; n = 4, ^*^*p* < 0.05.


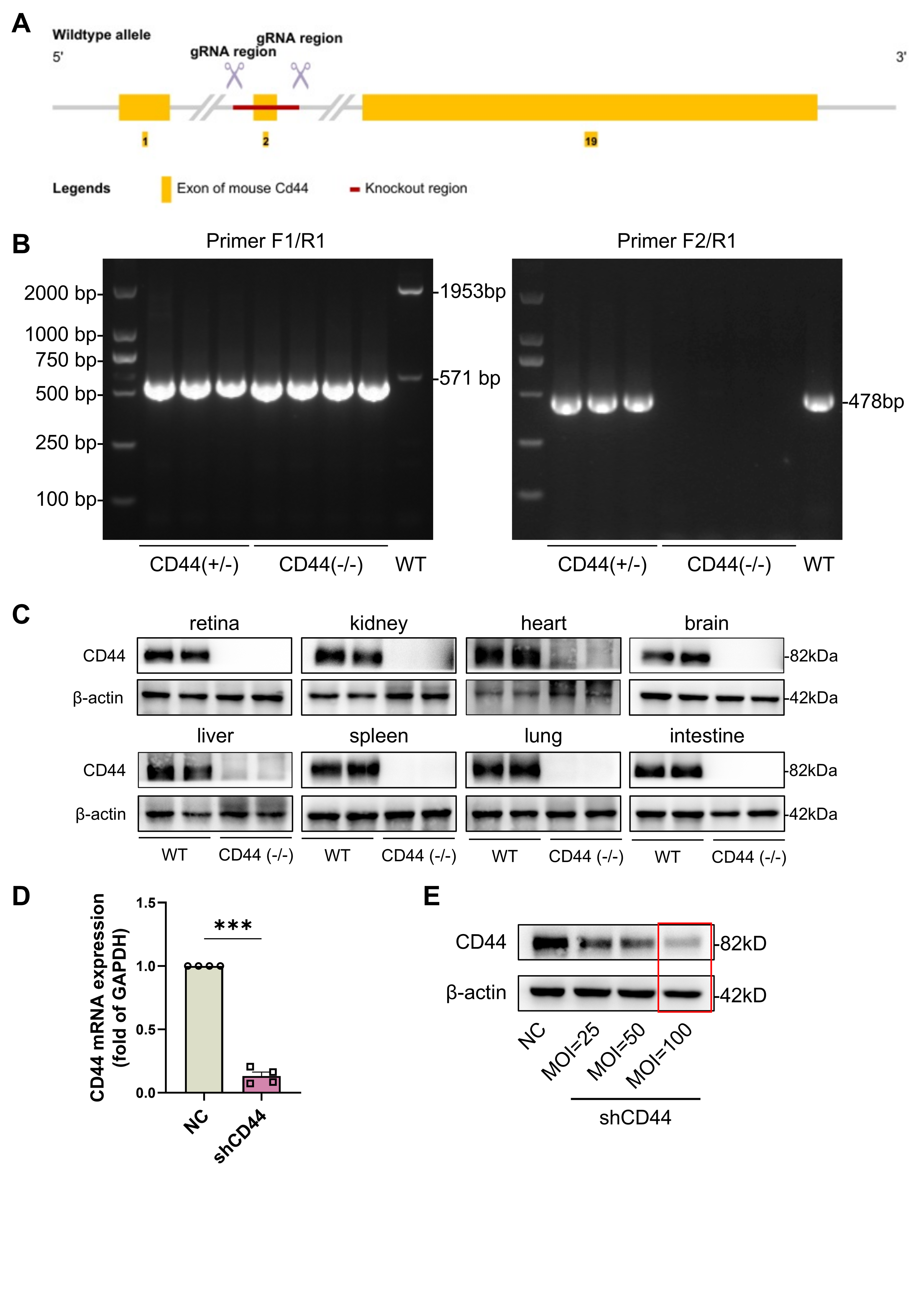


**Figure S5.** Knockout/knockdown of CD44 at in vivo and ex vivo levels. (A) Schematic diagram of the construction strategy of CD44 knockout mice. (B) DNA gel electrophoresis results of WT mice and CD44 knockout mice. (C) Protein expression of CD44 in various tissues and organs of WT mice and CD44 knockout mice. (D) mRNA level of CD44 after transfection of HUVECs with shCD44. Statistical analysis was performed using two-tailed unpaired t-test; n = 4, ^***^*p* < 0.001. (E) CD44 protein expression in endothelial cell after transfection with shCD44, and the red box indicates the optimal MOI (multiplicity of infection) for the use of shCD44.


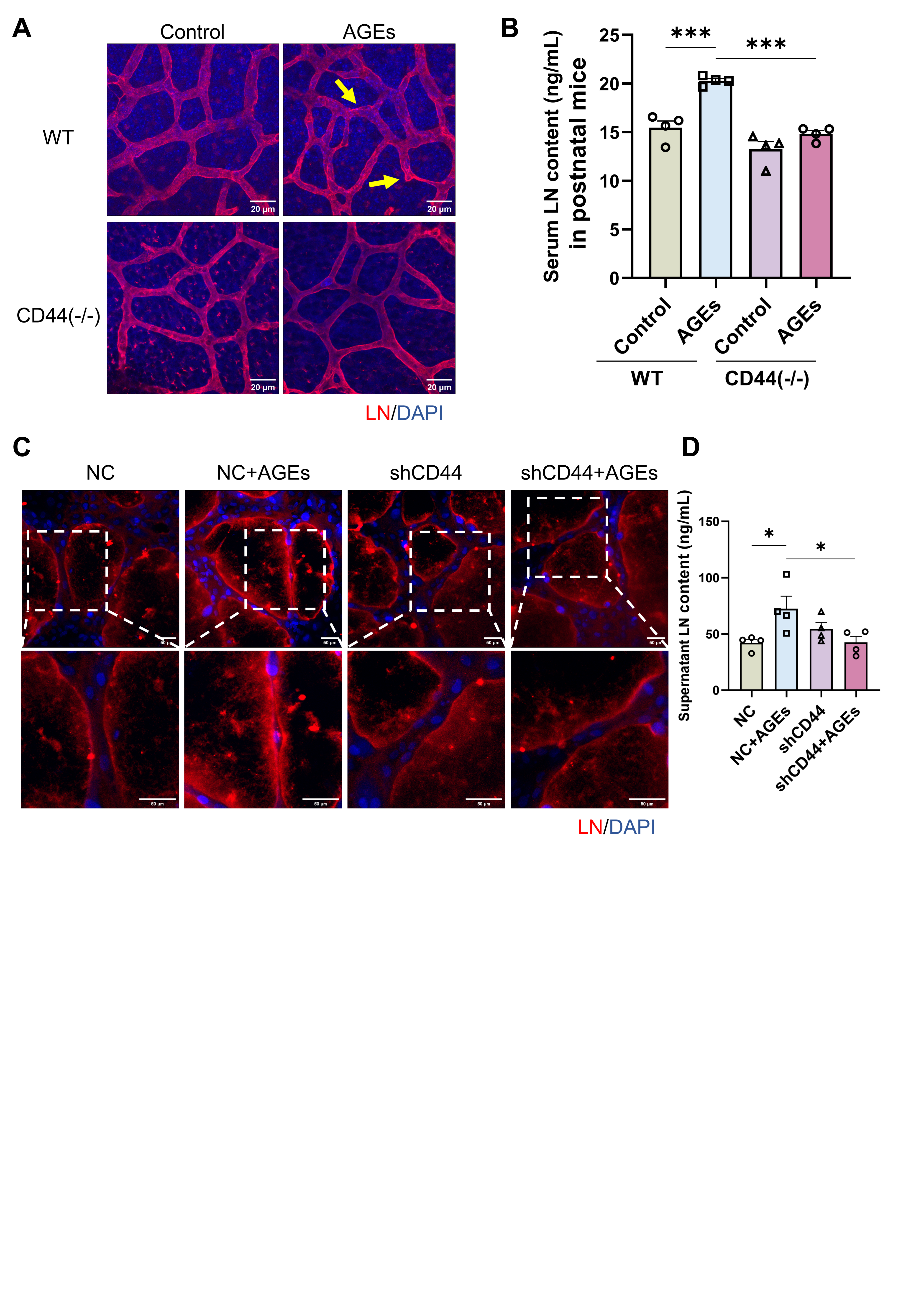


**Figure S6.** CD44 knockdown alleviated AGEs-induced maldistribution of LN in neonatal mice angiogenesis. (A) LN fluorescence staining in retina of AGEs-treated WT and CD44 knockout mice. (B) Serum LN content of WT and CD44 knockout mice treated with AGEs. Results were tested by one-way ANOVA and Turkey's multiple tests; n = 4, ^***^*p* < 0.001. (C-D) immunofluorescence staining of LN in the BM and LN content in the co-culture supernatant of shCD44-treated HUVECs with pericytes. Analyzed statistically using one-way ANOVA and Tukey's multiple comparison test; n = 4, ^*^*p* < 0.05.


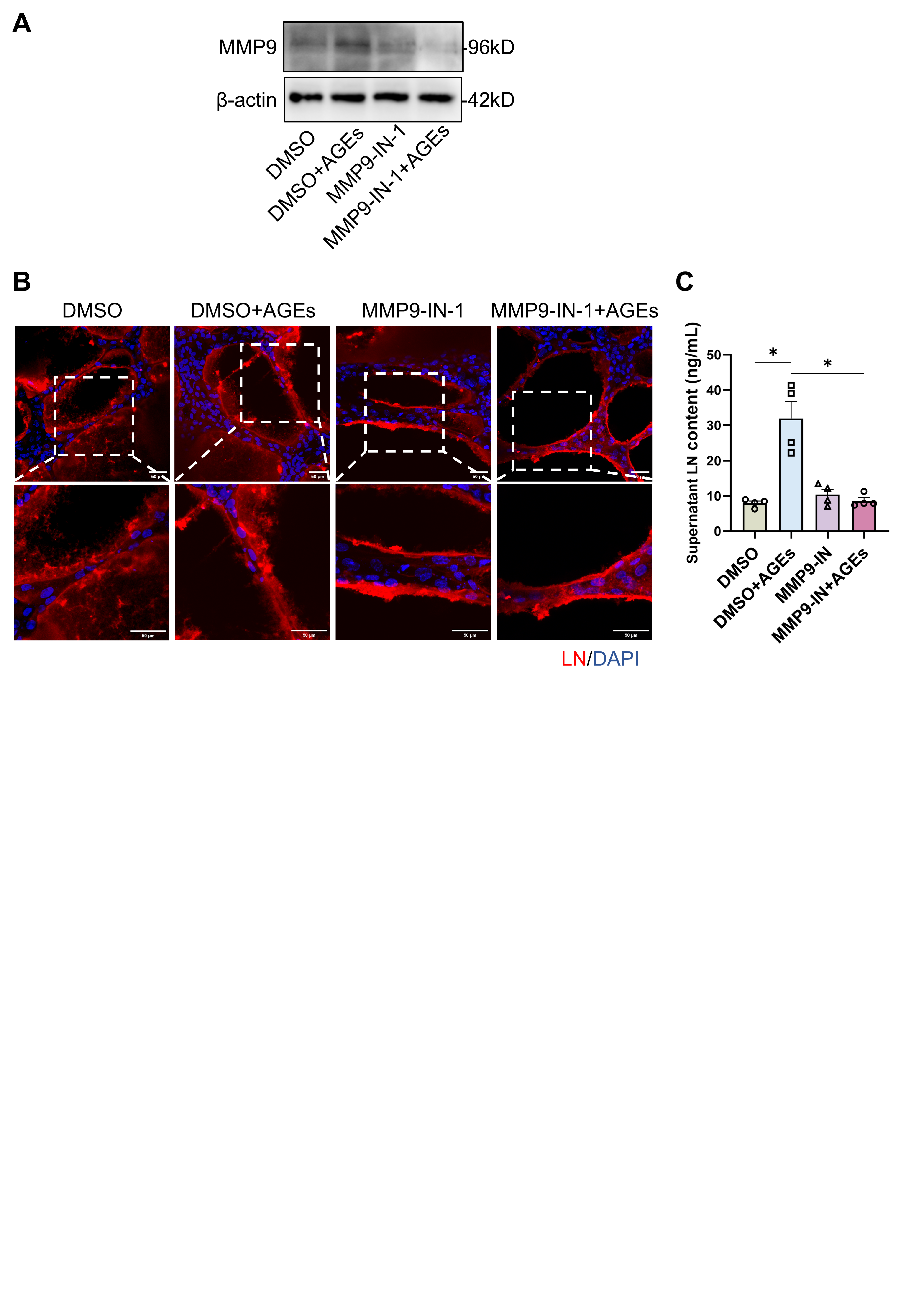


**Figure S7.** MMP9 inhibition affects the distribution of LN in the vascular basement membrane of HUVECs co-cultured with pericytes. (A) MMP9-IN-1 inhibits MMP9 protein expression in HUVECs; (B) LN fluorescence staining of MMP9-IN-1treated HUVECs co-cultured with pericytes to form tubules, with a scale of 50 μm; (C) LN content in supernatants of MMP9-IN-1-treated HUVECs co-cultured with pericytes. Results were statistically analyzed by one-way ANOVA and Dunnett's test, n = 4, ^*^*p* < 0.05.


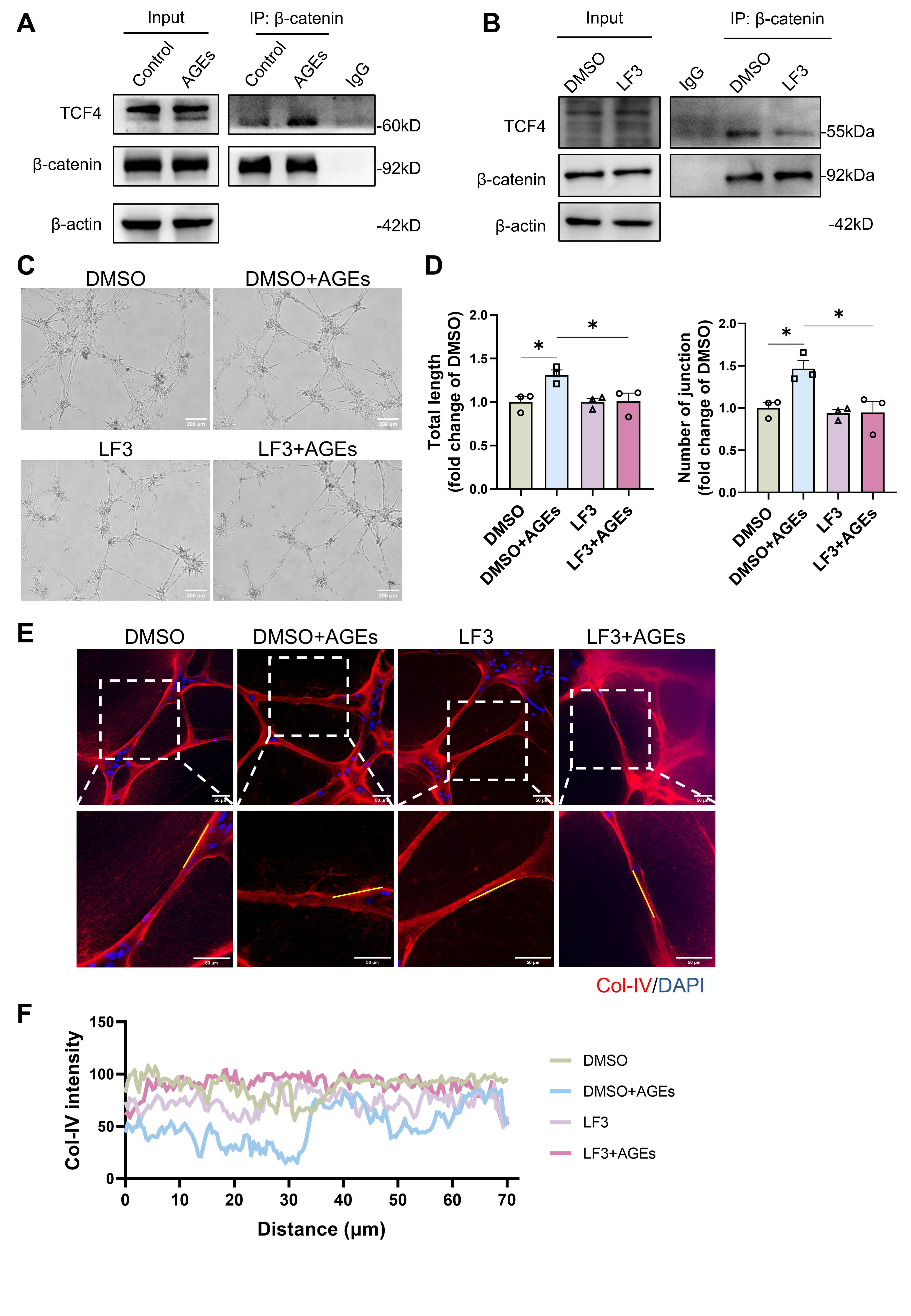


**Figure S8.** Interaction of β-catenin with TCF4 is involved in AGEs-induced angiogenesis and its BM structural abnormalities. (A) The results of co-immunoprecipitation of retinal tissue proteins in neonatal mice after 7 days of AGEs treatment. (B) Validation of the efficiency of LF3 antagonist in HUVECs. (C) Representative tube formation of LF3 treated HUVECs co-cultured with pericytes, with a scale of 200 μm. (D) The total length of tube formation and the number of branching nodes of LF3-treated HUVECs co-cultured with pericytes. The results were statistically analyzed by one-way ANOVA and Tukey's test, n = 3, ^*^*p* < 0.05. (E-F) Immunofluorescence staining and the distribution of Col-IV in LF3-treated HUVECs co-cultured with pericytes. Scale bar is 50 μm.
