# Supplementary material for: Increased CD44 Expression in Endothelial Cells Induced by Advanced Glycation End Products Leads to Insufficient Maturation of Angiogenesis
Source: J Cell Mol Med. 2026 Mar 16;30(6):e71088. doi: 10.1111/jcmm.71088 (PMC13097380; doi:10.1111/jcmm.71088)
Supplement: Supplementary file 2 — Table S1: Primer sequences for genotyping of CD44(−/−) mice. Table S2: List of antibodies. [file JCMM-30-e71088-s001.docx]

**Supplementary tables**

**Table S1.** Primer sequences for genotyping of CD44(-/-) mice

| Primer names | Primer sequence (5’-3’) |
| --- | --- |
| Forward primer 1 | AAGTGATAAGGCAAGGGGTAACAC |
| Reverse primer 1 | CCATAGCAGCATAGAAGCTCAAC |
| Forward primer 2 | TGAAACATGCAGGTAAGAGAGCAG |

**Table S2.** List of antibodies

| Antibody | Source and Identifier | Application |
| --- | --- | --- |
| CD44 | Immunoway, YT6201 | WB 1:1000 |
| CD44 | Abcam, ab119348 | IF 1:100 |
| Collagen Ⅳ | Immunoway, YT1024 | WB 1:1000  IHC 1:200 |
| Collagen Ⅳ | Abcam, ab6586 | IF 1:100 |
| Laminin | Sigma-Aldrich, L9393 | IF 1:100 |
| β-catenin | Immunoway, YM3403 | WB 1:1000 |
| β-catenin | Proteintech, 66379-1-Ig | IP 1:50 |
| TCF4 | CST, 2569 | WB 1:1000 |
| CD44ICD | Prepared by Immunoway  (Antigen peptide sequence: DQFMTADETRNLQNVDMKIGV) | WB 1:50 |
| MMP9 | ABclonal, A0289 | WB 1:750 |
| β-actin | Beijing Ray Antibody Biotech, RM2001 | WB 1:10000 |
| Goat anti-Mouse IgG(H+L)-HRP | Beijing Ray Antibody Biotech, RM3001 | WB 1:10000 |
| Goat anti-Rabbit IgG(H+L)-HRP | Beijing Ray Antibody Biotech, RM3002 | WB 1:10000 |
| Goat Anti Mouse IgG(H+L) (AbFluor 488) | Immunoway, RS3208 | IF 1:200 |
| Goat Anti Mouse IgG(H+L) (AbFluor 594) | Immunoway, RS3608 | IF 1:200 |
| Goat Anti Rabbit IgG(H+L) (AbFluor 594) | Immunoway, RS3611 | IF 1:200 |
| Goat Anti Rat IgG (DyLight 488) | Immunoway, RS23240 | IF 1:200 |
| DAPI | Bestbio, BB-4133 | IF 1:100 |
